# Observed fingerprint of global warming exposes model biases in regional climate attribution

**DOI:** 10.1126/sciadv.aed1506

**Published:** 2026-08-14

**Authors:** Hasi Aru, Dirk Olonscheck, Jochem Marotzke, Chao Li

**Affiliations:** ^1^ Max Planck Institute for Meteorology, Hamburg 20146, Germany.; ^2^ Earth and Society Research Hub, University of Hamburg, Hamburg 20146, Germany.

## Abstract

Disentangling externally forced signals from internal variability in observed surface air temperature (SAT) is essential for reliable regional climate detection and attribution. Although climate model simulated patterns broadly align with observed global warming, persistent regional discrepancies, compounded by the limitation of a single observational realization, impede robust regional attribution. We identify the observation-based SAT externally forced signals and internal variability, which allows us to compare the observed SAT patterns with the simulated counterparts from seven CMIP6 single-model initial-condition large ensembles. Perfect-model tests confirm robust large-scale partitioning, while indicating reduced fidelity where internal variability dominates. Our results suggest that models tend to overestimate emergence timescales in externally forced regions but underestimate them in internal variability–dominated regions. In several internal variability–dominated regions, the forced signal remains undetectable in observations. While Arctic warming is increasingly dominated by external forcing, internal variability has slowed its rate in recent decades. In internal variability–dominated regions, we identify modest yet consistent externally forced contributions, with observed cooling trends and sign flips primarily driven by internal variability. These findings help to resolve long-standing regional attribution puzzles, highlight critical model limitations, and carry fundamental implications for regional climate projections, attribution, and jurisprudence.

## INTRODUCTION

Although global warming is unequivocal (*1*), substantial uncertainties persist in detecting and attributing regional surface air temperature (SAT) trends (*2*, *3*). These uncertainties stem largely from two sources: (i) incomplete representation of regional processes in climate models, and (ii) the confounding effects of pronounced internal climate variability (*4*–*9*). Despite recent methodological advances, there is still no comprehensive, observation-based global fingerprint of externally forced warming that allows systematic model-observation comparison.

While a general alignment between simulated and observed SAT trend patterns exists, systematic discrepancies persist at regional scales. Notable examples include the difference in simulated versus observed Arctic amplification, attributed either to model biases in representing the forced Arctic/global temperature response or an underrepresentation of internal variability (*10*–*14*). These issues are exacerbated by systematic challenges in simultaneously simulating realistic Arctic sea-ice loss alongside plausible global warming (*15*–*17*). Moreover, climate models consistently struggle to reproduce observed cooling trends in the eastern tropical Pacific and Southern Ocean (*6*, *18*–*22*), and uncertainties in underlying mechanisms have led to inconsistent simulations of the timing and amplitude of the North Atlantic warming hole (*23*–*29*). These regions, where persistent model-observation mismatches remain an open problem and undermine confidence in regional-scale attribution and projection, are referred to hereafter as “contested regions.”

Widely used detection and attribution approaches could be compromised by regional model-observation mismatches. In particular, optimal fingerprinting relies on model-derived “fingerprints” to distinguish forced signals from internal variability (*4*, *30*–*32*), yet biases in models limit accuracy. Although optimal fingerprinting has proven effective at the global scale (*33*), regional model-observation mismatches may limit its reliability in attribution and projection. Alternative strategies, such as detrending, temporal filtering (*34*, *35*), or timescale separation techniques (*36*–*38*), avoid model dependence by assuming that forced changes evolve more slowly than internal variability. However, recent studies demonstrate that internal variability can substantially influence regional SAT trends even on multidecadal timescales (*39*–*42*). As a result, traditional temporal separation methods risk confounding low-frequency internal variability modes with externally forced signals, such as Atlantic multidecadal variability (*43*), limiting the reliability of the methods in regional attribution contexts.

To circumvent the limitations, there are a few methods that use global-scale forced warming as a proxy for external forcing. In practice, the globally averaged annual mean surface air temperature (GSAT) derived from the ensemble mean of a single-model initial-condition large ensemble (LE) is often used to represent externally forced signals. Linear regression techniques that use ensemble mean GSAT as a predictor have proven effective for isolating regional decadal internal variability, particularly in the context of Atlantic multidecadal variability (*44*). More recently, a Global Warming Index–based linear regression method was used to differentiate global-scale versus regional-scale drivers and local feedback mechanisms influencing observed land surface temperature trends (*45*). Complementing these efforts, the Forced Component Estimation Statistical Method Intercomparison Project systematically evaluated the methods for estimating long-term (1950–2022) externally forced sea surface temperature trends, including a regression approach that uses the ensemble-mean GSAT from 50 Community Earth System Model, version 2 (CESM2) large-ensemble members to separate external forcing from internal variability (*46*). However, these approaches are largely limited to specific diagnostics and do not explicitly derive a globally consistent, observation-based spatial fingerprint of externally forced warming.

**Fig. 1.**
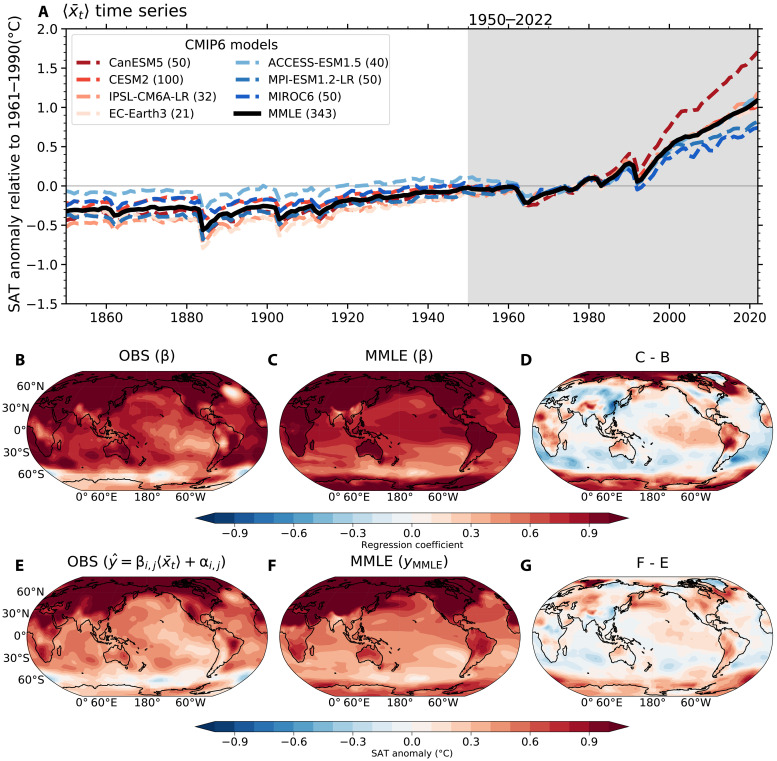
Illustration of the OBS-LPS framework. (**A**) Time series of GSAT anomalies (1850–2022) from seven LEs (colored dashed lines) and their multimodel LE mean (MMLE; black solid). (**B**) Observed SAT “fingerprint” pattern obtained by regressing annual mean SAT anomalies (1850–2022) at each grid point onto the MMLE GSAT time series $\langle {\bar{x}}_{t}\rangle$, representing the externally forced response in observations. (**C**) MMLE-derived fingerprint, computed by applying the same regression procedure across ensemble members using single-LE GSAT and then averaging across models (see Materials and Methods). (**D**) Difference between MMLE and observed fingerprints. (**E**) OBS-LPS reconstructed externally forced annual-mean SAT anomalies in observations, and (**F**) corresponding externally forced SAT anomalies in the MMLE, each averaged over 1993–2022. (**G**) Difference between MMLE and observed SAT anomalies, shown in (F) and (E), highlighting spatial biases in simulated warming patterns. All SAT anomalies are defined relative to the 1961–1990 climatology.

To fill this gap, we develop a diagnostic framework, “observed linear pattern scaling” (OBS-LPS). This method partitions observed SAT trends into externally forced and internal variability components by adopting ordinary least squares regression of observed SAT anomalies at each grid point onto the GSAT time series derived from the multimodel mean of seven CMIP6 LEs (hereafter MMLE; Fig. 1A). Unlike prior approaches limited to specific regions, OBS-LPS provides a global, observation-based fingerprint of forced warming. To avoid reliance on a single trend length and the attendant risk of aliasing internal variability into the estimated externally forced response (*39*–*41*), we (i) diagnose a time-invariant fingerprint using the longest feasible calibration period (1850–2022; Fig. 1B) and (ii) apply this “observed global fingerprint” to partition trends over multiple retrospective periods ranging from 10 to 73 years, with primary analyses focusing on 1950–2022.

We further introduce a previously unknown metric—the emergence timescale—defined as the observational record length (anchored retrospectively at 2022) required for the externally forced signals to distinctly and persistently emerge from internal variability. This metric provides an observation-based assessment of regional emergence timescales, facilitating robust model-observation comparison and offering a stringent test of the ability of climate models to realistically represent regional emergence behavior.

By isolating externally forced fingerprints directly from global observations, the OBS-LPS framework delivers a comprehensive, observation-based global warming fingerprint. This allows robust, like-for-like comparison with climate model simulations and helps resolve persistent systematic discrepancies between models and observations, especially in contested regions.

## RESULTS

### Diagnosed observed fingerprint and its implications

We outline the OBS-LPS framework, designed to partition observed SAT anomalies into externally forced and internal variability components. Motivated by the robust evidence that the latest generation of climate models can adequately reproduce the GSAT (*1*, *47*), we construct a univariate linear regression framework in which the MMLE GSAT anomalies serve as a high signal-to-noise regressor for the externally forced signals (Fig. 1 and figs. S1 and S2; Materials and Methods). At each grid point, observed SAT anomalies are regressed onto this MMLE GSAT anomaly time series to extract the externally forced “fingerprint.”

The resulting observed fingerprint reveals spatially inhomogeneous warming, with strong amplification over the Northern Hemisphere land and high-latitude oceans, weaker warming over the eastern Pacific, and cooling in the Southern Ocean and the midlatitude North Atlantic (Fig. 1B). While the MMLE-simulated fingerprint broadly captures the land-ocean warming contrast (Fig. 1C), it fails to reproduce Southern Ocean cooling, the NAWH, and the tropical Pacific gradient (Fig. 1D). The difference pattern suggests that the MMLE overestimates Arctic warming while it underestimates midlatitude land area warming (Fig. 1D). We display the most recent 30-year (1993–2022) constructed SAT anomalies in observations and MMLE to illustrate the contemporary impact of these biases (Fig. 1, E and F), wherein MMLE overpredicts warming in regions such as the Barents-Kara Seas, the Kuroshio Extension, the tropical eastern Pacific, and the subpolar North Atlantic, while it underpredicts warming in midlatitude Southern Hemisphere (Fig. 1G).

**Fig. 2.**
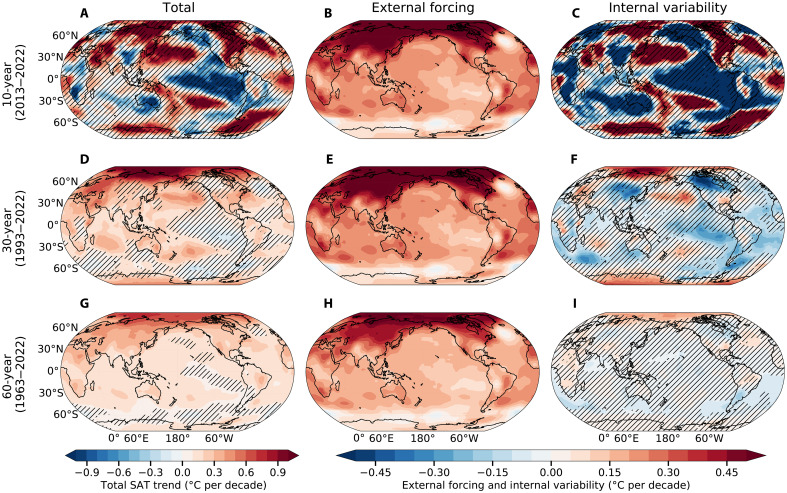
Partitioning of observed SAT trend patterns into external forcing and internal variability. (**A**) Total, (**B**) external forcing, and (**C**) internal variability observed SAT trend patterns for the most recent [(A) to (C)] 10-year (2013–2022), (**D** to **F**) 30-year (1993–2022), and (**G** to **I**) 60-year (1963–2022) periods, respectively, by regressing observed local anomalies against the 1850–2022 MMLE GSAT time series. Hatched regions denote statistically insignificant trends at the 95% confidence level, as determined by the nonparametric Mann-Kendall trend test.

We adopt the OBS-LPS method as a diagnostic framework to quantitatively partition SAT trend patterns into external forcing and internal variability components (Fig. 2). The derived externally forced SAT patterns show substantial warming in the Arctic and over Northern Hemisphere land, as well as stronger warming in the Indian Ocean and the western Pacific compared to the central-eastern Pacific (Fig. 2, B, E, and H). Cooling patches in the Southern Ocean and muted warming in the subpolar North Atlantic are also evident, and consistent across different timescales (Fig. 2, B, E, and H). Significant warming in externally forced SAT is offset by internal variability–driven cooling in the central-eastern tropical Pacific, while internal variability amplifies SAT changes in polar regions (Fig. 2, C and F). These patterns remain robust when applying a 10-year Butterworth low-pass filter to remove interannual variability, confirming that the partition primarily reflects low-frequency variability rather than high-frequency noise (figure not shown). In light of the predominance of existing research focusing on the satellite era (1979–2022), we have undertaken a comparative analysis of satellite trend patterns for this particular period (figs. S5). The resulting externally forced and internal variability contributions are qualitatively similar and closely resemble the 30-year trend partitions. The counteracting effect of internal variability in cooling the North Atlantic, southeastern Pacific, and Southern Ocean reinforces the notion of its critical role in shaping observed regional SAT patterns.

### Method validation and model skill assessment

We now demonstrate the robustness of the OBS-LPS method by leveraging LEs, treating each ensemble member as a pseudo-observation to validate method performance. Within each LE, the ensemble mean represents the externally forced response, while the deviation from the ensemble mean represents internal variability. We apply OBS-LPS to each member to reconstruct externally forced and internal variability components and then compare the reconstructed patterns against the known ensemble-wise externally forced signal and ensemble spread. This controlled evaluation framework allows us to assess how well the method recovers the true underlying patterns. A reliable method should reproduce externally forced and internal variability patterns that closely resemble the ensemble mean and ensemble spread, respectively.

**Fig. 3.**
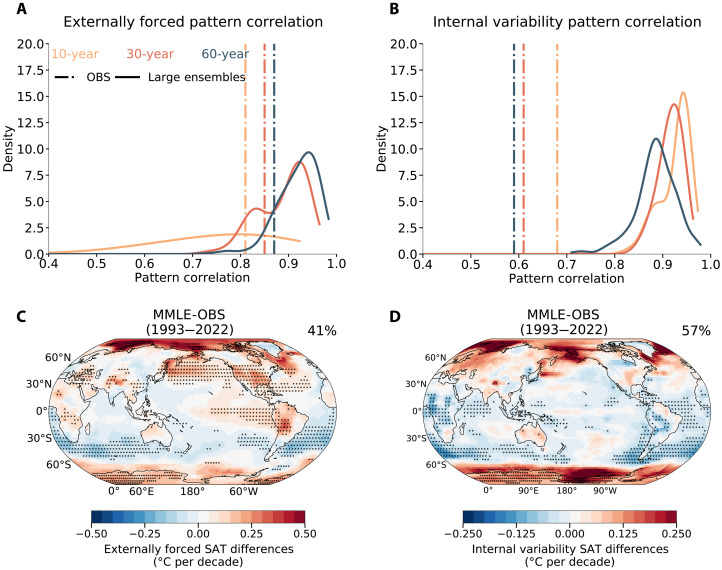
Evaluation of the OBS-LPS method and the performance of LEs in capturing externally forced and internal variability SAT trend patterns. (**A**) Pattern correlations between ensemble member–simulated externally forced SAT patterns and the LEs ensemble mean pattern; (**B**) pattern correlations for internal variability SAT patterns between individual ensemble members and the LE ensemble spread (7 LEs, 343 members; see Materials and Methods and table S1). Solid lines show correlations among simulated patterns derived via OBS-LPS; dash-dot lines show pattern correlations of the diagnosed observed patterns versus MMLE-simulated patterns. Results are displayed for 10-year (2013–2022, orange), 30-year (1993–2022, red), and 60-year (1963–2022, blue) trends. (**C**) Spatial difference between MMLE-simulated and observed externally forced SAT patterns for the 30-year period (1993–2022). (**D**) Same as (C), but for internal variability (trend standard deviation). Stippling in (C) and (D) indicates regions where MMLE-OBS differences exceed the 95% OBS-LPS error range estimated from perfect-model experiments. Percentages in (C) and (D) denote the area-weighted RMS of the LE-OBS difference pattern normalized by the area-weighted RMS of the observed pattern, i.e., the global bias-amplitude fraction.

We quantify the reconstruction skill using spatial pattern correlation and root mean square error (RMSE; see Materials and Methods). Since pattern amplitude varies with trend length τ, the raw RMSE is not directly comparable across τ. To enable cross-timescale comparisons, we report the normalized RMSE, ${\text{nRMSE}}^{r}\left(\tau \right)={\text{RMSE}}^{r}\left(\tau \right)/\sigma^{\text{ENS}}\left(\tau \right)$, where $\sigma^{\text{ENS}}\left(\tau \right)$ is the spatial standard deviation of the ensemble mean reference pattern for specific τ. Higher correlation and lower nRMSE indicate higher fidelity in partitioning externally forced and internal variability components. Across multidecadal trends, correlations between OBS-LPS reconstructions and the ensemble mean/spread references are typically > 0.8 with low nRMSE (generally ≲0.6), whereas skill degrades for 10-year windows (Fig. 3 and fig. S10). These consistently high correlations and low nRMSE support the robustness of OBS-LPS in recovering the large-scale externally forced and internal variability trend patterns, while indicating that some residual regional differences remain, particularly on short timescales.

To assess model fidelity in reproducing observed SAT trend patterns, we compare the diagnosed observed externally forced and internal variability components with those simulated by the LEs. We compute pattern correlations between the observed externally forced and internal variability SAT fields and the corresponding ensemble mean and spread. However, these “observed” pattern correlations (hereafter “observed externally forced/internal variability SAT”) deviate substantially from the distribution of analogous simulated ensemble mean/spread pattern correlations across ensemble members (hereafter “simulated distribution”) (Fig. 3, A and B). The observed externally forced SAT resides at the lower bound of the simulated distribution, while the observed internal variability SAT falls entirely outside it. These discrepancies indicate that LEs struggle to reproduce the spatial characteristics of both the externally forced and internal variability–driven SAT trend patterns.

The partitioned observed patterns provide a systematic basis for “like-for-like” assessment of LEs’ skill in reproducing externally forced and internal variability SAT trend patterns (see Materials and Methods; Fig. 3 and figs. S8 to S10). Subtracting the observed 30-year (1993–2022) externally forced and internal variability SAT trends from the MMLE-simulated counterparts yields the spatial MMLE-observation (MMLE-OBS) differences (Fig. 3, C and D). The MMLE overestimates externally forced signals in the polar regions (Arctic and Antarctic), the midlatitude North Pacific, the tropical eastern Pacific, and the subpolar North Atlantic, while it underestimates these signals in the midlatitude Southern Hemisphere (Fig. 3C). For internal variability, the MMLE exhibits stronger amplitudes in the subpolar Arctic and Antarctic but weaker amplitudes in the tropical Pacific and subtropical Southern Hemisphere (Fig. 3D). The MMLE-OBS biases explain 41 and 57% of the variance in the diagnosed observed externally forced and internal variability SAT trend patterns, respectively.

To account for uncertainty in the OBS-LPS framework, we estimate a grid point–dependent error envelope using perfect-model experiments (Materials and Methods). Regions where model-observation differences exceed this uncertainty range are stippled, indicating discrepancies that are unlikely to be explained by methodological uncertainty. In contrast, nonstippled regions include both areas of close agreement and areas where differences are not statistically distinguishable from OBS-LPS uncertainty. For the externally forced component (Fig. 3C), robust MMLE-OBS differences are concentrated in the Arctic, the midlatitude North Pacific, parts of the tropical eastern Pacific, and sectors of the Southern Hemisphere midlatitudes. In these regions, the MMLE-OBS discrepancies exceed the estimated OBS-LPS uncertainty and therefore provide strong evidence for genuine model-observation differences in the externally forced SAT response. By contrast, in regions without stippling, the apparent MMLE-OBS differences are either small or remain within the expected uncertainty range of the OBS-LPS method and thus cannot be robustly attributed to model bias alone. For the internal variability component (Fig. 3D), robust discrepancies are most evident along the Antarctic margin and across parts of the Southern Ocean and subtropical Southern Hemisphere oceans, with additional localized signals in the tropical Atlantic–eastern Pacific sector. In these regions, the MMLE either overestimates or underestimates the amplitude of internal variability relative to observations by an amount that exceeds the inferred OBS-LPS error envelope. This suggests that at least part of the MMLE-OBS mismatch in internal variability reflects genuine deficiencies in the simulated variability field rather than methodological uncertainty. At the same time, the absence of stippling over other regions indicates that some MMLE-OBS differences in internal variability are not distinguishable from OBS-LPS uncertainty and should therefore be interpreted more cautiously.

Substantial intermodel spread emerges in LE performance, with indications of a link to equilibrium climate sensitivity (ECS) (figs. S8 and S10). LEs with lower ECS exhibit higher pattern correlations and closer agreement with observations, whereas LEs with higher ECS exhibit lower pattern correlations and tend to overestimate warming in the Northern Hemisphere mid and high latitudes (fig. S8). LE-observation biases in externally forced patterns vary by model: Lower-ECS LEs (e.g., MIROC6 and MPI-ESM1.2-LR; fig. S8, C and D) show smaller discrepancies than the higher-ECS LEs (e.g., IPSL-CM6A-LR and CanESM5; fig. S8, G and I). For internal variability, models differ in their ability to replicate observed amplitudes across different timescales (figs. S9 and S10), although the overall bias pattern is broadly similar across LEs regardless of the ECS (fig. S9). The seven LEs analyzed span ECS values of roughly 2.6° to 5.6°C; three fall within the Intergovernmental Panel on Climate Change Sixth Assessment Report likely ECS range (2.5° to 4.0°C) (*48*), while several exceed these ranges. We therefore caution that any apparent ECS-skill relationship should be interpreted prudently, given the limited sample size and potential confounding by other structural model differences. These findings reveal persistent limitations in climate models and highlight the need to improve the simulation of both externally forced and internal variability SAT patterns, particularly concerning regional fidelity.

### Observed versus simulated emergence timescale

Conventional time-of-emergence studies project forward to estimate when a forced climate signal first rises above background internal climate variability (*49*–*52*). Here, we introduce a complementary backward-looking metric—the emergence timescale—which quantifies, relative to a fixed anchor year (2022), how many years of past observations are required for the externally forced signal to become detectable and remain robust against internal variability at all longer timescales (see Materials and Methods). This retrospective definition provides a spatially resolved diagnostic for assessing the historical detectability of externally forced signals and their separation from internal climate variability.

**Fig. 4.**
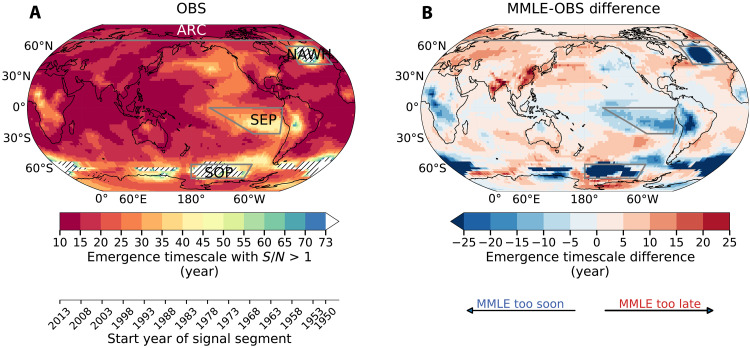
Observed and simulated emergence timescale of SAT trends anchored at the year 2022, spanning 1950–2022. (**A**) Emergence timescale of observed externally forced SAT trends, defined as the shortest backward-looking trend length (anchored at 2022) for which the signal-to-noise ratio (S/N)(τ) exceeds 1 and remains above this threshold at all longer trend lengths (i.e., monotonic emergence; see Materials and Methods). Colored shading reflects the emergence timescale (years), while the overlaid secondary *x* axis indicates the corresponding start year of each signal segment. White areas indicate no emergence within the 10- to 73-year window, and hatching marks grid points that either show no emergence or fail the monotonicity test (Materials and Methods). (**B**) Difference between MMLE-derived and observed emergence timescales. Positive values (red shading) indicate that the MMLE exhibits delayed emergence relative to observations (i.e., overestimation of the emergence timescale), whereas negative values (blue shading) indicate earlier emergence relative to observations (i.e., underestimation of the emergence timescale). Gray-outlined boxes mark four key areas referenced in Fig. 5: ARC (66.5°N poleward), NAWH (42°N to 60°N, 50°W to 10°W), SEP, SOP (70°S to 55°S, 180°W to 100°W). Patterns of emergence timescales for individual LE are presented in fig. S12.

Analysis of observational data reveals that externally forced SAT signals typically emerge from internal variability within ∼30 to 35 years. However, pronounced regional heterogeneity is evident (Fig. 4A). Rapid emergence (10 to 15 years) occurs in the Arctic, Indo-Pacific warm pool, tropical North Atlantic, Southern Atlantic, and some coastal areas. Moderate emergence timescales (30 to 45 years) are evident in the northern and tropical eastern Pacific and much of the Southern Ocean, while forced signals remain undetectable in the NAWH and parts of the Southern Ocean, even over the 73-year observed record.

The broad MMLE-OBS discrepancies in externally forced and internal variability SAT patterns (Fig. 3, C and D) prompt further examination of the emergence timescales in seven LEs (Materials and Methods; fig. S12). All LEs reveal a distinct poleward gradient: rapid emergence in the tropics and progressively delayed emergence toward the poles, in particular across the Southern Hemisphere (fig. S12). Higher-ECS models respond more rapidly to external forcing, leading to an earlier global detectability around 30-year trend lengths (fig. S12H). The comparison between observed and MMLE-derived emergence timescales highlights that MMLE shows delayed emergence in the Arctic, mid-to-high latitude continental regions (e.g., North America, Europe, and Southeast Asia), and along Antarctic coastlines, while simulating earlier than observed emergence in the eastern equatorial Pacific, patches of the Southern Ocean, and the North Atlantic subpolar gyre (Fig. 4B). Individual LEs vary in their ability to reproduce the observed emergence timescales: Lower-ECS LEs more closely resemble observations (e.g., MPI-ESM1.2-LR and ACCESS-ESM1.5; fig. S12, C, and D), whereas the higher-ECS LEs diverge (e.g., CESM2 and CanESM5; fig. S12, G and H). A stricter detectability threshold S/N>2 leaves spatial patterns and model-observation contrast qualitatively unchanged; absolute timescales shift later, but preserved regional rankings and MMLE-OBS differences persist (figures not shown). Collectively, these results suggest that LEs systematically produce a skewed depiction of emergence, driven by deficiencies in representing internal variability and externally forced responses.

### Understanding contested regions

**Fig. 5.**
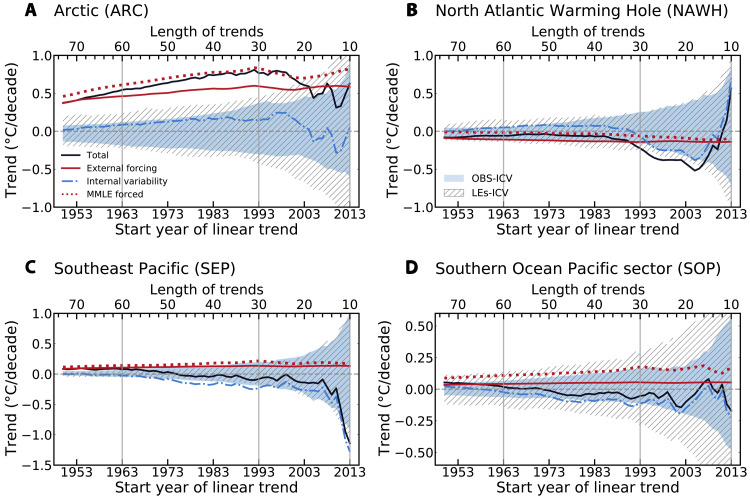
Evolution of total, externally forced, and internal variability SAT trends in four key regions (1950–2022, anchored at 2022). (**A**) Arctic, (**B**) North Atlantic warming hole, (**C**) SEP, and (**D**) SOP; regions are defined in Fig. 4 (Materials and Methods). For each panel, regional-mean linear trends are computed over backward-looking windows ending in 2022, with the start year advanced annually from 1950 to 2013 (top axis indicates the corresponding trend length). Black solid lines show total SAT trends; red solid lines show OBS-LPS diagnosed externally forced trends; and blue dash-dot lines show the diagnosed internal variability component. Light blue shading denotes the 5th to 95th percentile range of observed internal variability estimates, derived from all overlapping trend samples within 1850–2022. The red dotted line shows the MMLE-simulated forced trend. Hatched shading denotes the 5th to 95th percentile range of MMLE internal variability diagnosed from seven CMIP6 LEs using an ensemble-residual approach (text S1). Gray vertical lines mark the specific trend intervals highlighted in Figs. 2 to 4. ICV, internal variability.

To help resolve the ongoing debate on the relative contributions of external forcing versus internal variability to regional SAT changes, we examine four contested regions: the Arctic, the North Atlantic warming hole, the southeast Pacific (SEP), and the Southern Ocean Pacific (SOP) sectors (see Materials and Methods for definitions of regional indices). In the Arctic, externally forced warming dominates the total observed trend and emerges distinctly against internal variability for trend lengths of only 15 years and more (Fig. 5A). This aligns with the rapid emergence of Arctic warming shown in Fig. 4A. While total Arctic warming continues at a high rate, internal variability has slowed the warming pace over the past two decades. Despite decadal changes in the sign and magnitude of internally driven Arctic trends, externally forced warming remains the dominant contributor to long-term Arctic warming. This conclusion is robust to choices of trend anchoring: backward-looking trend partitions anchored in 2013 likewise show external forcing as the dominant contributor to Arctic warming (fig. S14). With the partitioned externally forced Arctic trends, it becomes evident that the MMLE overestimates the externally forced Arctic warming rates (Figs. 3C and 5A). Our findings complement the previous studies that highlight the substantial role of internal climate variability in recent Arctic SAT changes (*13*, *14*), while explicitly demonstrating the timescale dependence of its influence: Internal variability modulates the externally forced signals on decadal windows, whereas the externally forced component dominates at multidecadal timescales.

In the subpolar North Atlantic, the historically observed cooling, known as the NAWH (*25*, *53*, *54*), has been widely linked to external forcing, although considerable model uncertainty remains (*28*, *55*–*57*). Our analysis demonstrates that externally forced cooling consistently influences NAWH SAT trends and becomes dominant for trend lengths of 30 years and more (Fig. 5B). Internal variability, however, plays a crucial role in driving short-term change, reversing the sign of total trends over the past 30 years. Internal variability initially amplified cooling before shifting to drive a recent spike in warming, signaling a potential regime transition from cooling to warming. To test sensitivity to the choice of endpoint, we repeat the backward-looking trend partitioning with trends anchored in 2013 (fig. S14). For longer trend lengths anchored in 2013 (≥20-year), NAWH shows notable total warming, driven largely by internal variability (fig. S14). Conversely, short-term trends continue to show cooling due to a predominant externally forced cooling effect (fig. S14). The endpoint dependence highlights the temporal sensitivity of SAT trend attribution in the NAWH and suggests the challenge of separating externally forced signals from internal variability.

In the SEP and SOP, observed cooling trends have diverged from climate model simulations (*6*, *18*, *19*, *21*, *22*, *58*–*60*). We find modest but consistent contributions from externally forced warming over all timescales considered (Fig. 5, C and D). This contrasts with earlier suggestions that external forcing reinforced regional cooling (*60*, *61*). Internal variability is the dominant driver of shorter-term trends in these regions, particularly in the SEP, where a strong cooling excursion occurred over the past 10 to 15 years. In the SOP, internal variability inflates total observed SAT trends, complicating the disentanglement of human and internal contributions. For trend lengths of 30 years or longer, long-term trends in both regions are consistent; results are qualitatively similar whether trends are anchored in 2022 (Fig. 5) or in 2013 (fig. S14). The coherent cooling between SEP and SOP further highlights the role of Southern Ocean teleconnections in modulating regional variability (*22*, *59*). Observed internal variability across four regions closely aligns with the margins of the internal variability distribution, especially on longer timescales (Fig. 5 and fig. S14). This highlights the dominant role of observed internal variability in shaping regional SAT patterns and raises concerns about biases in climate models when simulating regional internal variability (fig. S9).

To assess how well the models reproduce the observed regional SAT trend partitions, we compare the OBS-LPS–diagnosed regional SAT trends with the externally forced signals and internal variability estimated from the ensemble mean and ensemble-residual approach of LEs, respectively (Fig. 5; see Materials and Methods and text S1). Consistent with the spatial pattern discrepancies identified earlier (Fig. 3, C and D), the MMLE systematically overestimates both externally forced signals and internal variability in the Arctic and NAWH (Fig. 5, A and B). The MMLE adequately simulates internal variability in the SEP but overestimates internal variability in the SOP (Fig. 5, C and D). The coherence across pattern, emergence, and regional trend diagnostics supports the internal consistency of the OBS-LPS partitioning and simultaneously reveals model biases in simulating regional internal variability. These findings reinforce the value of the OBS-LPS framework for improving regional attribution, diagnosing model-observation mismatches, and informing targeted improvements in climate model fidelity.

## DISCUSSION

Our OBS-LPS method has allowed us to identify externally forced and internal variability in observed surface warming patterns, facilitating a direct, like-for-like comparison with climate model simulations. We help reconcile long-standing observation-model mismatches, particularly in regions characterized by slowly emerging forced signals, by explicitly identifying where simulated forced responses remain inadequate and where models reliably capture observed changes. By quantifying the relative contribution of external forcing and internal variability across space and time, our analysis helps resolve key attribution ambiguities in contested regions exhibiting prolonged emergence timescales, thus bridging previously conflicting interpretations of the role of external forcing in shaping regional climate trends.

The reduction in total Arctic warming over the past two decades is primarily due to internal variability–driven cooling, counteracting the long-term externally forced warming trend. Likewise, the intermittent cooling or diminished warming in the SEP is chiefly controlled by internal variability. Over the past decade alone, internal variability has driven the total cooling trend excursion in the SEP, exceeding the expected amplitude of internal variability, signaling a potential regime shift toward weaker cooling or even potential warming in the future. A pressing consideration remains whether the total observed SAT trends in the SOP sector will transition to externally forced warming or continue to be dominated by internal variability–driven cooling. Given the prolonged emergence timescales of externally forced signals in the SOP sector (Fig. 4), externally forced warming will remain obscured by the background noise of internal climate variability in the near future, emphasizing the need for long-term monitoring and refined model simulations to resolve their relative influence in shaping regional SAT trends.

Our method quantifies the emergence timescale for externally forced surface warming from observations, pinpointing persistent regional model biases. While simulated and observed externally forced SAT patterns generally align, systematic regional biases persist, particularly in contested regions. These discrepancies highlight the need for rigorous model evaluation to improve future climate projections. By refining our understanding of external forcing, our results have substantial implications for enhancing assessments of regional climate-change potential, which is critical for evaluating climate impacts and guiding mitigation strategies.

Despite the robustness of the OBS-LPS method, several caveats merit attention. First, the assumption of linearity between global SAT and local responses, combined with the time-invariant nature of the derived observational fingerprint, may limit our ability to capture dynamically evolving, circulation-driven SAT patterns (*62*). Second, by using an aggregated GSAT predictor, our approach cannot distinguish the distinct contributions of individual forcing agents (*63*), such as the evolving regional influence of anthropogenic aerosol emissions. Third, the observational fingerprint, and thus the resulting externally forced and internal variability partitions, is inevitably affected by observational uncertainty, incomplete early coverage, and potential artefacts introduced by infilling in sparsely observed regions. Future developments should aim to extend the framework to incorporate evolving forcing patterns, circulation adjustments, and forcing-specific predictors, thereby enabling a more detailed and physically grounded regional attribution.

In addition, perfect-model experiments reveal that the performance of the OBS-LPS framework is not spatially uniform (fig. S13). Regions such as the southeastern Pacific exhibit systematic estimation errors across models and ensemble members, indicating reduced fidelity in areas characterized by weak signal-to-noise ratios or spatially complex internal variability. By quantifying the full distribution of estimation errors using large ensembles, we construct a grid point–dependent uncertainty envelope and explicitly incorporate this uncertainty into the interpretation of model-observation differences. This uncertainty-aware framework distinguishes between robust discrepancies and differences that are not statistically distinguishable from methodological uncertainty. It prevents overattribution of model biases and establishes a more conservative basis for regional climate attribution. While the method reliably captures large-scale patterns, regional interpretations should therefore be made with caution, particularly in internal variability–dominant regions.

In conclusion, the OBS-LPS method provides an observation-based and physically grounded framework for partitioning observed SAT trends into externally forced and internal variability, offering a valuable tool for assessing regional externally forced climate impacts. The derived externally forced warming patterns expose unresolved model biases, underscoring the need to improve model fidelity in capturing the spatial structure of observed climate change. Furthermore, the partitioning allows an evaluation of the consistency among fingerprints derived from formal detection and attribution methods, refining regional climate projections, and advancing our understanding of SAT trend dynamics.

## MATERIALS AND METHODS

### Observational and model data

We use three century-long blended near-surface temperature records, each of which combines land 2-m air temperature over continents with sea surface temperature over the oceans: HadCRUT5.0.2 [the Met Office Hadley Centre/Climatic Research Unit global surface temperature dataset version 5; (*64*)], NOAAGlobalTemp [the National Oceanic and Atmospheric Administration Global Surface Temperature Dataset; (*65*)], and Berkeley Earth Surface Temperature [the Berkeley Earth Land/Ocean Temperature Record; (*66*)]. All three datasets provide monthly anomalies from 1850 to the present, constructed by merging land-based air temperature measurements with marine sea surface temperatures, thereby yielding a globally complete “blended” surface temperature field. To quantify the variability in temperature trends, we incorporate output from six LEs under the auspices of CMIP6 that archived monthly historical SAT data (“tas”), namely, ACCESS-ESM1.5 (*67*), CanESM5 (*68*), EC-Earth3 (*69*), IPSL-CM6A-LR (*70*), MIROC6 (*71*), and MPI-ESM-1-2-LR (*72*). We also consider the CESM version 2 large ensembles, CESM2 (*73*), wherein the surface temperature is “TREFHT.” These models were selected based on their availability of more than 20 ensemble members, which allows for robust statistical characterization of internal variability across spatial and temporal scales (*74*). Within a given LE, all ensemble members are subjected to the same set of historical external forcings, anthropogenic (e.g., greenhouse gases and aerosols) and natural (e.g., solar variability and volcanic eruptions), offering unique insights into internal climate variability through their ensemble spread, initialized with marginally differing initial conditions. As the historical simulations end in 2014, we extended the CMIP6 model data to 2022 using Shared Socioeconomic Pathway 2-4.5 (SSP2-4.5) experiments, and the CESM2 data were extended to 2022 using SSP3-7.0. All data are first compiled on a regular 2° by 2° horizontal resolution by bilinear interpolation to enable comparison between observational data and different climate models. Temperature anomalies are calculated relative to the 1961–1990 baseline. Model details are summarized in table S1. To minimize dependence on any single model, for multimodel analyses, we first compute the mean of each LE, then average those seven means to form the multimodel LE mean (MMLE), yielding a robust estimate of externally forced responses of SAT across models. Unless noted otherwise, HadCRUT5 provides the observational reference in the main analysis.

### Observed linear pattern scaling method

We base our analysis on the premise that the GSAT from the multimodel mean of seven CMIP6 single-model initial-condition large ensembles (MMLE) provides the best a priori estimate of the externally forced SAT response to all forcings (*44*). We thus adopt the MMLE-derived GSAT time series as a robust proxy for the externally forced driver to derive observed fingerprints. To delineate externally forced warming patterns in observed SAT anomalies, we use the MMLE GSAT time series from historical simulations spanning 1850–2022, denoted as $\langle {\bar{x}}_{t}\rangle$. The ensemble mean GSAT for year *t* is calculated as a cosine-latitude-weighted global average of SAT (Fig. 1A). At each grid point (i,j), we apply the OBS-LPS method, a univariate linear regression relating observed local SAT anomalies $y_{\mathrm{ij}}\left(t\right)$ to the MMLE-derived GSAT time series $\langle {\bar{x}}_{t}\rangle$. This regression yields an empirical estimate of the externally forced component ${\hat{y}}_{\mathrm{ij}}\left(t\right)$, representing the expected externally forced response in the observed SAT anomalies, while the residuals ε from this reconstruction are interpreted as internal climate variability (i.e., intrinsic fluctuations unexplained by external forcing)

$$y_{\mathrm{ij}}\left(t\right)={\hat{y}}_{\mathrm{ij}}\left(t\right)+\varepsilon_{\mathrm{ij}}\left(t\right)$$ (1)

$${\hat{y}}_{\mathrm{ij}}\left(t\right)=\beta_{\mathrm{ij}}\langle {\bar{x}}_{t}\rangle +\alpha_{\mathrm{ij}}$$ (2)

where the spatiotemporal vectors *y* denote the total SAT anomalies, which consist of the externally forced $\hat{y}$ and internal variability ε spatiotemporal vectors (Eq. 1). Equation 2 provides the empirical separation of these two components, where β is the regression coefficient obtained by regressing total observed SAT anomalies at each grid point onto the MMLE-simulated GSAT time series $\langle {\bar{x}}_{t}\rangle$ (shown in both Fig. 1A and fig. S1). The regression coefficients quantify the degree of local observed SAT response attributable to globally averaged external forcing, in this context also retrieved as the observed fingerprint of SAT (compare fig. S2A). In addition, Eq. 2 delineates α, a spatially varying intercept pattern (fig. 7B), which represents the baseline offset that aligns each observed local time series with the GSAT-scaled forced component. Figure S2D shows the diagnosed externally forced component of observed SAT anomalies over the most recent 30-year period, isolated from the influence of internal variability, thus yielding an estimate of the externally forced signals $\hat{y}$. Compared with the raw SAT anomalies (fig. 7C), the residual variations can be ascribed to internal variability (fig. S2E).

We assess the sensitivity of the method to the choice of both observational record and climate model by applying the ensemble mean GSAT time series from each LE to the regression model (see figs. S3, S4, S6, and S7). The partitioned SAT patterns are qualitatively consistent across the different observational datasets (Fig. 2 and figs. S3 and S4). Similarly, the partitioned SAT patterns remain robust across different climate models, regardless of the LE used as the GSAT predictor. Pattern correlations between each individual-LE–based partition and the MMLE-derived counterpart exceed 0.99 for the externally forced SAT patterns and 0.83 for the internal variability SAT patterns (see figs. S6 and S7).

To verify that the 10-year partitioning is not unduly influenced by interannual variability, we repeated the analysis after applying a 10-year Butterworth low-pass filter to the observed SAT anomalies. This filter removes fluctuations shorter than a decade while retaining the longer-term signal relevant to this study. The total, externally forced, and internal variability SAT trend patterns derived from the filtered data (figure not shown) closely match those from the unfiltered data (compare Fig. 2). This insensitivity confirms that the partition is robust and primarily reflects low-frequency variability rather than high-frequency noise.

To quantify the evolving contributions of the externally forced signals and internal variability to regional SAT trends, we define four key regional indices as follows: (i) the ARC index, calculated as the area-averaged SAT trend over 66.5°N to 90°N, following (*10*); (ii) the NAWH index, defined as the difference between the mean SAT over the subpolar gyre region (${\text{SAT}}_{\text{sg}}$; 42°N to 60°N, 50°W to 10°W) and the Northern Hemisphere mean sea surface temperature (${\text{SST}}_{\text{NH}}$), i.e., $\text{NAWH}={\text{SAT}}_{\text{sg}}-{\text{SST}}_{\text{NH}}$, aligning with previous definitions (*25*); (iii) the SEP index, calculated as the area-averaged SAT trend over a specified trapezoidal region; and (iv) the SOP index, defined as the area-averaged SAT trend over 70°S to 55°S, 180°W to 100°W. These regions are outlined in Fig. 4.

### Diagnosing externally forced and internal variability SAT trends: Observations and LEs

We analyze observed temperature patterns for three characteristic periods: the most recent 10-year (2013–2022), 30-year (1993–2022), and 60-year (1963–2022) periods. The externally forced and internal variability components of SAT anomalies are obtained from Eqs. 1 and 2. Trends in both components are then estimated using Theil-Sen Slope estimator (*75*, *76*), a nonparametric method that estimates the median rate of change over time and is robust to outliers. Trend significance is assessed using the nonparametric Mann-Kendall test (*77*). Trends that are not significant at the 95% confidence level are indicated with slanted hatching.

To characterize internal variability from a single observational realization, we compute the distribution of local trend estimates across overlapping running windows of varying length τ∈[10,73] years. We use the full observational record from 1850–2022 to maximize the sample size. For each τ, we construct a running-trend field by fitting a linear trend over an admissible τ-year window for all start years $t_{0}$ in [1850,2022−τ], shifting the window 1 year at a time. This yields, at each grid point (i,j), an ensemble of trend samples representing the range of the internal variability.

We assume that internal variability $\varepsilon_{\mathrm{ij}}\left(t\right)$ follows a normal distribution $N\left(0,\sigma_{\mathrm{ij}}^{2}\right)$, where $\sigma_{\mathrm{ij}}$ denotes the standard deviation of internal variability, calculated as

$${\hat{\sigma }}_{\mathrm{ij}}\left(\tau \right)=\sqrt{\frac{1}{173-\tau -1}\sum_{t_{0}=1850}^{2022-\tau }{\left[b\left({\tilde{x}}_{\mathrm{ij}},t_{0},\tau \right)-\bar{b}({\tilde{x}}_{\mathrm{ij}},\tau )\right]}^{2}}$$ (3)

where ${\hat{\sigma }}_{\mathrm{ij}}\left(\tau \right)$ is the standard deviation of internal variability SAT trends $b\left({\tilde{x}}_{\mathrm{ij}},t_{0},\tau \right)$ for variable $\tilde{x}$ at grid point (i,j) over the time interval $\left[t_{0},t_{0}+\tau \right]$ years, while $\bar{b}\left({\tilde{x}}_{\mathrm{ij}},\tau \right)$ is the mean of $b\left({\tilde{x}}_{\mathrm{ij}},t_{0},\tau \right)$ with respect to the initial time $t_{0}$. The term 173−τ−1 corresponds to the total number of τ-year running windows within the 173-year period (1850–2022). The uncertainty ranges are summarized by the 5th to 95th percentile of the sampled internal variability trends. Externally forced trend values exceeding this range indicate the timescale at which forced signals are inferred to dominate observed regional SAT trends. This approach yields estimates of temperature variability patterns across multiple timescales, with all running windows anchored to end in 2022, and provides a reference distribution for assessing recent trend evolution.

To characterize forced SAT trends in each LE, we calculate the linear trend of the ensemble mean SAT anomalies of each LE and of the MMLE. For isolating the internal variability, we subtract the ensemble mean SAT anomalies from each individual realization, yielding residual internal variability. We then compute linear trend patterns of these residuals for each ensemble realization and characterize the spread of internal variability trends by calculating the sample standard deviation across realizations for each time interval and grid point

$${\hat{\sigma }}_{\mathrm{ij}}^{\text{LE}}\left(\tau \right)=\sqrt{\frac{1}{N-1}\sum_{n=1}^{N}{[b\left({\tilde{x}}_{\mathrm{ij}}^{n},\tau \right)-{\bar{a}}_{\mathrm{ij}}\left(\tau \right)]}^{2}}$$ (4)

$${\bar{a}}_{\mathrm{ij}}\left(\tau \right)=\frac{1}{N}\sum_{n=1}^{N}b\left({\tilde{x}}_{\mathrm{ij}}^{n},\tau \right)$$ (5)

Here, ${\hat{\sigma }}_{\mathrm{ij}}^{\text{LE}}\left(\tau \right)$ is the sample standard deviation of internal variability trends $b\left({\tilde{x}}_{\mathrm{ij}}^{n},\tau \right)$ of the residual component in variable $\tilde{x}$, at grid point (i,j) for the *n*-th ensemble member, and ${\bar{a}}_{\mathrm{ij}}\left(\tau \right)$ is the ensemble mean of $b\left({\tilde{x}}_{\mathrm{ij}}^{n},\tau \right)$.

To evaluate how well the LEs reproduce the partitioned observational patterns, we use spatial pattern correlation and RMSE. The RMSE quantifies the discrepancy between N samples of LE predictions $\hat{y}$ relative to the samples of *N* observations y. For each trend length τ∈[10,73] years, we compute end-fixed trends over the interval [2022−τ+1, 2022] and obtain two corresponding spatial trend fields: the observationally estimated forced trend pattern $b_{\mathrm{ij}}^{\text{OBS}}\left(\tau \right)$ and the model-predicted forced trend pattern $b_{\mathrm{ij}}^{\text{MMLE}}\left(\tau \right)$. The spatial RMSE for a given τ is defined as

$$\text{RMSE}\left(\tau \right)=\sqrt{\frac{1}{N_{\Omega }\left(\tau \right)}\sum_{\left(i,j\right)\in \Omega \left(\tau \right)}{\left[b_{\mathrm{ij}}^{\text{MMLE}}\left(\tau \right)-b_{\mathrm{ij}}^{\text{OBS}}\left(\tau \right)\right]}^{2}}$$ (6)

where Ω(τ) denotes the set of grid points with valid data in both patterns, and $N_{\Omega }\left(\tau \right)$ is the number of such grid points. To facilitate comparison across trend lengths with different signal amplitudes, we also report the nRMSE

$$\text{nRMSE}\left(\tau \right)=\frac{\text{RMSE}\left(\tau \right)}{\sigma^{\text{OBS}}\left(\tau \right)}$$ (7)

with $\sigma^{\text{OBS}}\left(\tau \right)$ is the spatial standard deviation of $b_{\mathrm{ij}}^{\text{OBS}}\left(\tau \right)$ over Ω(τ). Area-weighted spatial pattern correlations are computed over the same domain. Results for internal variability patterns are calculated analogously using the corresponding observed and LE fields.

### Method validation with LEs

To assess the robustness of the OBS-LPS partitioning, we performed the analysis entirely within each CMIP6 LE. For every ensemble member, we (i) fit the ordinary least squares regression model described in Eq. 2 using the GSAT time series from that member’s ensemble mean as the predictor, and (ii) computed externally forced and internal variability SAT trends for three characteristic periods (10-, 30-, and 60-year windows). In each LE, the ensemble-mean SAT pattern represents the forced response, while the ensemble spread characterizes internal variability. We evaluate the method’s skill by evaluating pattern correlation statistics, comparing the forced SAT pattern extracted from each member with the corresponding ensemble mean, and comparing the reconstructed internal variability pattern (standard deviation, as described in Eq. 3) with the ensemble spread pattern.

Across the seven CMIP6 LEs (343 members in total), Taylor diagrams indicate strong agreement between individual members and ensemble mean trend patterns for both the externally forced and internal variability components (fig. S10; see text S1). For the forced component, member patterns cluster at high pattern correlations (typically ≳ 0.9) with normalized standard deviations generally close to unity, implying that members share a highly consistent spatial fingerprint and comparable spatial-contrast amplitude relative to the ensemble mean forced pattern. Centered nRMSE values are correspondingly small, indicating limited residual pattern mismatch. For the internal variability component, members likewise exhibit high correlations with the ensemble-mean internal variability pattern and modest centered RMSE, with the main spread arising from differences in amplitude (normalized standard deviation) rather than changes in spatial structure. Together, these diagnostics show that the OBS-LPS framework yields stable and physically coherent partitions of externally forced and internal variability trend patterns across ensembles.

We evaluate how accurately the OBS-LPS method recovers the true externally forced and internal variability SAT trend patterns within a single realization of an LE, for which the true forced response is approximated by the LE ensemble mean. Specifically, for each model and each end-fixed sliding-window length τ∈[10,73] years, we compute SAT trends over the interval [2022−τ+1, 2022] and obtain two spatial trend fields: (i) $b_{\mathrm{ij}}^{\text{ENS}}\left(\tau \right)$, the model “true” forced trend pattern estimated as the LE ensemble mean trend pattern, and (ii) ${\hat{b}}_{\mathrm{ij}}^{r}\left(\tau \right)$, the forced (or internal variability) trend pattern estimated by OBS-LPS from an individual realization *r* (or, when applicable, the corresponding model-diagnosed internal variability pattern used as reference). Within the perfect-model framework, we quantify the estimation error of the OBS-LPS method at each grid point (i,j) as

$$\epsilon_{\mathrm{ij}}^{r}\left(\tau \right)={\hat{b}}_{\mathrm{ij}}^{r}\left(\tau \right)-b_{\mathrm{ij}}^{\text{ENS},r}\left(\tau \right)$$ (8)

where $b_{\mathrm{ij}}^{\text{ENS},r}\left(\tau \right)$ denotes the corresponding model “true” pattern: the ensemble mean for externally forced response and the residual-based internal variability reference for each realization. By pooling $\epsilon_{\mathrm{ij}}^{r}\left(\tau \right)$ across all realizations and models, we construct an empirical error distribution at each grid point and for each timescale τ. From this distribution, we estimate the 95% confidence interval $\left[\epsilon_{\mathrm{ij}}^{\text{low}}\left(\tau \right),\epsilon_{\mathrm{ij}}^{\text{high}}\left(\tau \right)\right]$, which provides a spatially explicit and data-driven measure of the OBS-LPS method uncertainty.

We then use this error envelope to assess the robustness of model-observation differences. For each grid point, the difference between the MMLE-simulated and OBS-LPS–derived trend, $\Delta_{\mathrm{ij}}\left(\tau \right)$, is considered robust only if it lies outside the corresponding 95% OBS-LPS error interval, i.e.

$$\Delta_{\mathrm{ij}}\left(\tau \right)<\epsilon_{\mathrm{ij}}^{\text{low}}\left(\tau \right)\;\text{or}\;\Delta_{\mathrm{ij}}\left(\tau \right)>\epsilon_{\mathrm{ij}}^{\text{high}}\left(\tau \right)$$ (9)

Otherwise, the discrepancy is interpreted as being within the expected uncertainty of the OBS-LPS method and therefore not statistically distinguishable from method-induced error. This framework enables a direct, grid point–level assessment of whether model-observation differences exceed the intrinsic uncertainty of the OBS-LPS partition.

To demonstrate the method’s robustness to the choice of GSAT predictor, we conducted a “leave-one-model-out” experiment. Treating all members of MPI-ESM1.2-LR as pseudo-observations, we repeat the regression using GSAT series from each of the six remaining LEs and from the MMLE as independent predictors. The externally forced and internal variability patterns recovered from these pseudo-observations correlate strongly with the MPI-ESM1.2-LR ensemble mean forced pattern and ensemble spread internal variability patterns, respectively (fig. S11). High correlations across all cross-model predictors indicate that OBS-LPS is largely insensitive to the specific GSAT predictor used and does not merely reproduce the spatial structure of the predictor model. These tests confirm that the OBS-LPS method provides a robust statistical partitioning of externally forced and internal variability contributions to SAT variations.

### Emergence timescale of SAT trends

Unlike conventional time-of-emergence analyses, which project forward to identify a future date when a forced signal first exceeds background variability, our backward-looking definition quantifies how many years of the historical record (retrospectively anchored at 2022) are required for robust detection. The signal *S* is defined as the magnitude of the linear trend in the externally forced component in observational SAT anomalies, computed over the backward-looking trend lengths anchored at 2022 and spanning lengths τ∈[10,73] years. The noise *N* is quantified as the sample standard deviation of the reconstructed internal climate variability, ${\hat{\sigma }}_{\mathrm{ij}}\left(\tau \right)$, as defined in Eq. 3. For each trend length τ, the signal-to-noise ratio(S/N)(τ) is calculated at each grid point to produce spatiotemporal maps of detectability.

We define the emergence timescale at a grid point as the shortest trend length for which (S/N)(τ)>1 and remains above 1 for all longer trend lengths, a condition we term “monotonic emergence.” Formally, let $\tau^{*}$ be the first trend length in the 10- to 73-year range such that

$$\frac{S}{N}\left(\tau^{*}\right)>1\;\text{and}\;\frac{S}{N}\left(\tau \right)>1,\;\forall \;\tau \geq \tau^{*}$$ (10)

For a stricter detectability criterion, we also identify the first trend length $\tau^{**}$ for which signal exceeds twice the noise

$$\frac{S}{N}\left(\tau^{**}\right)>2\;\text{and}\;\frac{S}{N}\left(\tau \right)>2,\;\forall \;\tau \geq \tau^{**}$$ (11)

The resulting $t^{*}$ is the emergence timescale at that grid point. The monotonicity criterion ensures that, once the forced signal emerges from internal variability, it remains detectable in all longer retrospective windows.

We apply the same protocol to each LE. For model m and member r, we compute ${\left(S/N\right)}_{m,r}\left(\tau \right)$ grid point–wise and diagnose the member-wise emergence time $\tau_{m,r}^{*}$ via Eq. 10 (and, where noted, via Eq. 11). The LE emergence map $\tau_{m}^{*}$ is taken as the member-median at each grid point; grid points where fewer than 85% of members satisfy the monotonic emergence are hatched to indicate weak within-model consensus (fig. S12). The MMLE emergence timescale pattern is then taken as the grid point–wise median across the seven LEs, in line with the IPCC AR6 practice of reporting multimodel medians as the central estimate. This unified framework facilitates observation-model comparisons and provides insight into the regional detectability of externally forced warming signals.
