# Supplementary material for: Observed fingerprint of global warming exposes model biases in regional climate attribution
Source: Sci Adv. 2026 Aug 14;12(33):eaed1506. doi: 10.1126/sciadv.aed1506 (PMC13475608; doi:10.1126/sciadv.aed1506)
Supplement: Supplementary file 1 [file sciadv.aed1506_sm.pdf]

Science Advances
AAAS

Supplementary Materials for

Observed fingerprint of global warming exposes model biases in regional climate attribution

Hasi Aru *et al.*

Corresponding author: Hasi Aru, josie.aruhasi@mpimet.mpg.de; Chao Li, chao.li@mpimet.mpg.de

Sci. Adv. **12**, eaed1506 (2026)
DOI: 10.1126/sciadv.aed1506

This PDF file includes:

Supplementary Text
Figs. S1 to S14
Table S1

Supplementary Text

Text S1. LE construction of regional internal variability

We estimate internal variability from the LEs using the standard residual approach, which isolates unforced fluctuations by removing the ensemble mean forced signal. For model m with realizations $r = 1, \ldots, n_m$,

$$\varepsilon_{m,r}(t, \mathbf{x}) = X_{m,r}(t, \mathbf{x}) - \overline{X}_m(t, \mathbf{x}), \tag{S1}$$

where $X_{m,r}$ is the SAT field in realization r and $\overline{X}_m(t, \mathbf{x})$ is the LE ensemble mean. Regional residual time series are computed as $\varepsilon^R_{m,r}(t) = \langle \varepsilon_{m,r}(t, \mathbf{x}) \rangle_{\mathbf{x} \in R}$, and end-fixed τ-year regional trend samples are

$$b^R_{m,r}(t_0, \tau) = \text{trend}\left(\left\{\varepsilon^R_{m,r}(t) : t \in [t_0,\, t_0 + \tau]\right\}\right), \tag{S2}$$

computed with the same trend estimator as in the observations (Theil–Sen). For each model m, the distribution of $\{b^R_{m,r}(t_0, \tau) : 1 \leq r \leq n_m\}$ defines a model–specific internal variability range via its 5th–95th percentile.

To construct the MMLE estimate without over-weighting large ensembles with many realizations (e.g., 100 members), we apply equal-model weighting. For each LE m, numbered from 1 to M and region R, we define internal variability residuals as deviations from the LE ensemble mean and compute end-fixed τ–year regional residual trends for each realization, yielding a sample $\{b^R_{m,r}(\tau) : 1 \leq r \leq n_m\}$ where n_m is the number of realizations for the model m. Pool all samples across models but assign each realization a weight inversely proportional to its ensemble size. The MMLE internal variability range for the region R for τ–year runs from the 5th to the 95th percentiles of the weighted samples,

$$Q_{0.05/0.95}\left(\left\{\frac{b^R_{m,r}(\tau)}{n_m} : 1 \leq m \leq M,\ 1 \leq r \leq n_m\right\}\right) \tag{S3}$$

where $Q_{0.05}$ and $Q_{0.95}$ denote the 5th and 95th percentiles, respectively. By construction, this procedure assigns equal total weight to each LE and prevents larger member ensembles from dominating the MMLE internal variability envelope.

Text S2. Spatial error metrics and LE skill

To quantify the mismatch between two spatial patterns for a fixed τ, we compute the grid–point RMSE for each realization r:

$$\mathrm{RMSE}^r(\tau) = \sqrt{\frac{1}{N_\Omega(\tau)} \sum_{(i,j)\in\Omega(\tau)} \left(\hat{b}^r_{ij}(\tau) - b^{\mathrm{ENS}}_{ij}(\tau)\right)^2}, \tag{S4}$$

where $\Omega(\tau)$ denotes the set of grid points with valid data in both patterns and $N_\Omega(\tau)$ is the number of such grid points. We compute $\mathrm{RMSE}^r(\tau)$ for each realization r and summarize its distribution across ensemble members using the median range.

Because the magnitude of trend patterns varies with τ, we also report a normalized RMSE:

$$\mathrm{nRMSE}^r(\tau) = \frac{\mathrm{RMSE}^r(\tau)}{\sigma^{\mathrm{ENS}}(\tau)}, \tag{S5}$$

where $\sigma_{\mathrm{ENS}}(\tau)$ is the spatial standard deviation of the reference pattern $b_{\mathrm{ENS}}(i, j; \tau)$ computed over the same set $\Omega(\tau)$:

$$\sigma^{\mathrm{ENS}}(\tau) = \sqrt{\frac{1}{N_\Omega(\tau)} \sum_{(i,j)\in\Omega(\tau)} \left(b^{\mathrm{ENS}}_{ij}(\tau) - \overline{b}^{\mathrm{ENS}}(\tau)\right)^2}, \tag{S6}$$

with

$$\overline{b}^{\mathrm{ENS}}(\tau) = \frac{1}{N_\Omega(\tau)} \sum_{(i,j)\in\Omega(\tau)} b^{\mathrm{ENS}}_{ij}(\tau). \tag{S7}$$

Note that Eq. (3) in the Materials and Methods characterizes variability across running windows at each grid point (varying t_0), whereas Eqs. (S4)–(S5) quantify the mismatch between two spatial patterns for an end–fixed window (varying (i, j)).

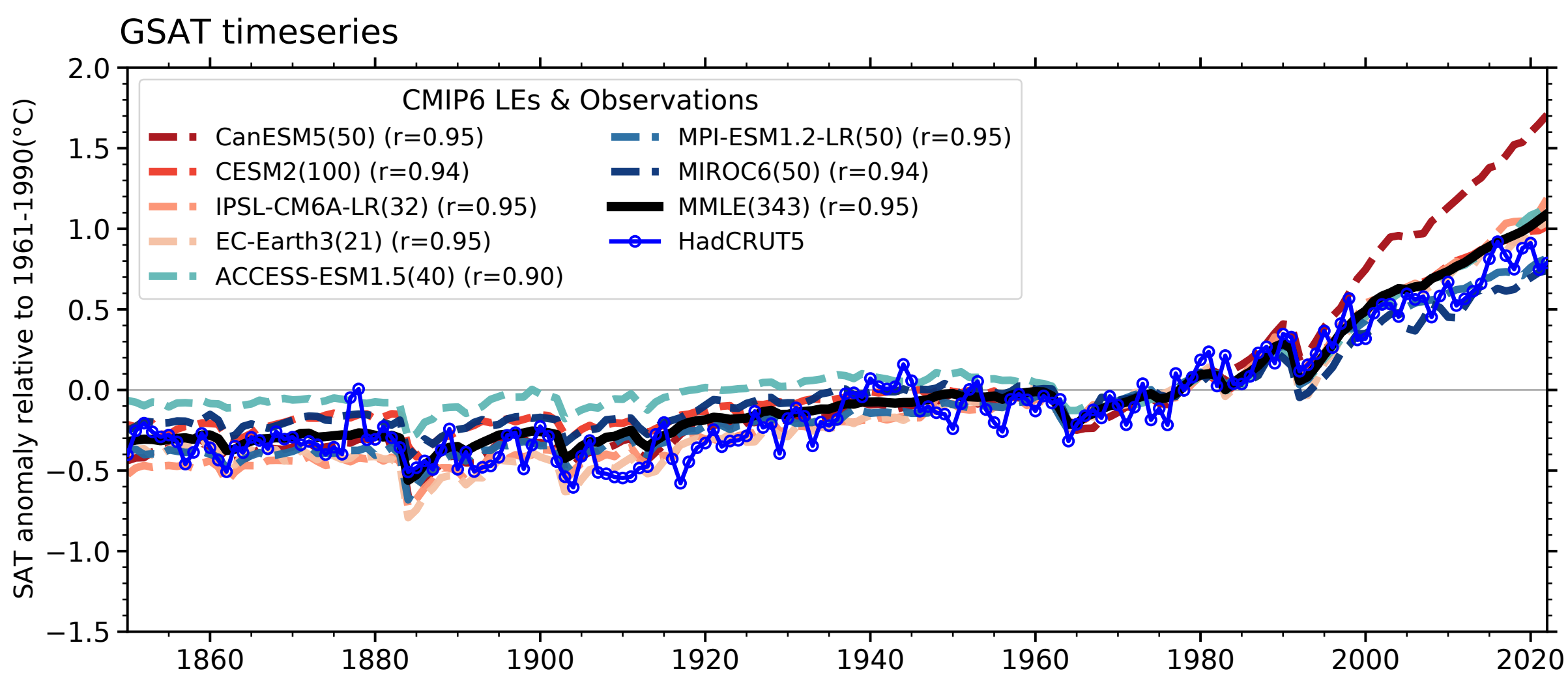

Fig. S1. CMIP6 large ensembles reproduce the observed GSAT evolution. Annual-mean GSAT anomalies (relative to 1961–1990) for HadCRUT5 (blue) and the ensemble mean GSAT of seven CMIP6 large ensembles (colored dashed lines) over 1850–2022; the MMLE mean is shown in black. Parentheses give ensemble size; r is the Pearson correlation of each modeled GSAT with HadCRUT5.

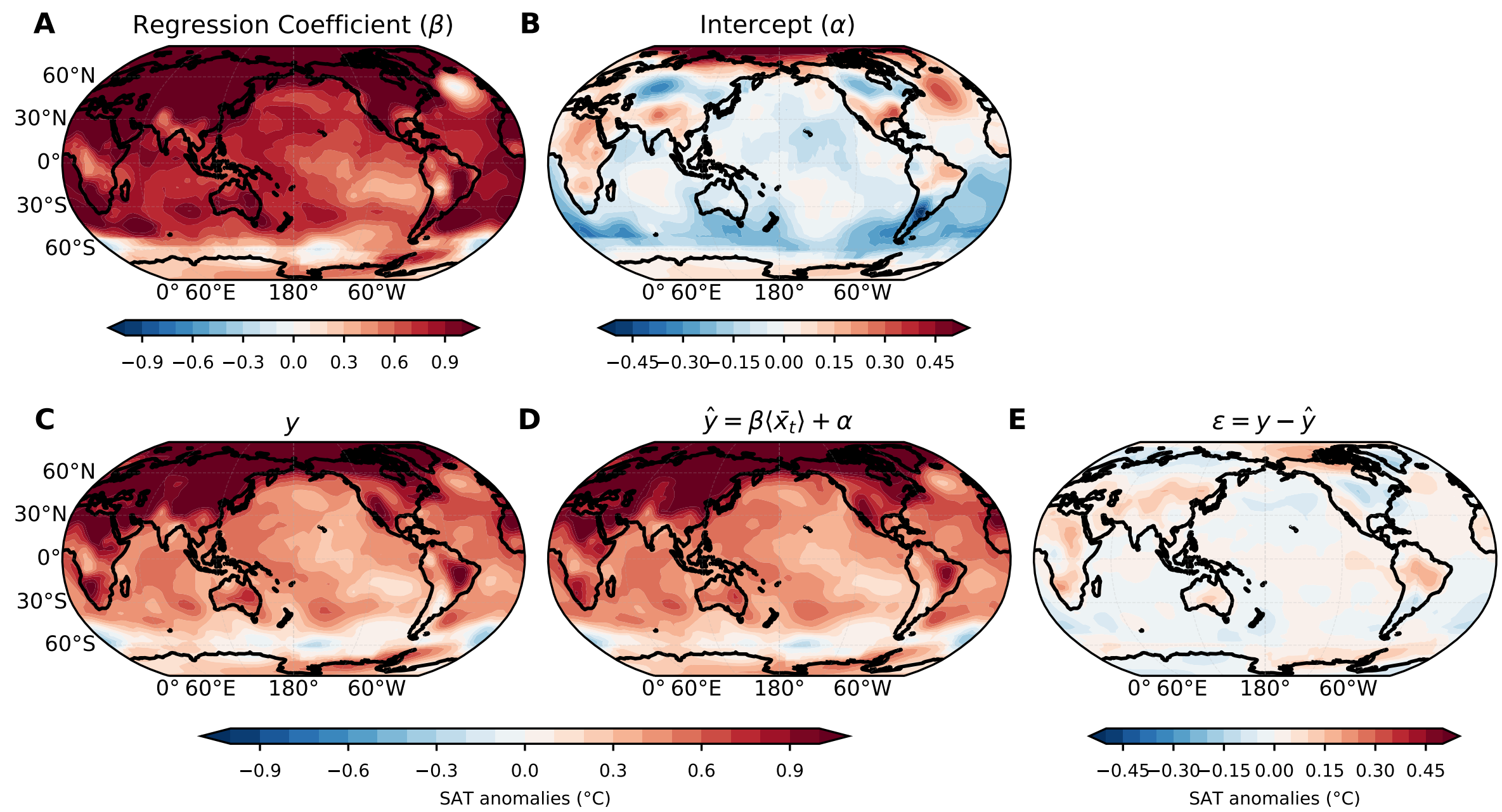

Fig. S2. Illustration of the methodology employed in this study. Spatial patterns of (**A**) the OBS–LPS fingerprint (regression slope, β) and (**B**) the regression intercept (α) obtained by regressing observed annual-mean SAT anomalies over 1850–2022 onto the MMLE GSAT time series $<\bar{x}_t>$. Spatial patterns of (**C**) raw observed SAT anomalies, (**D**) diagnosed externally forced SAT anomalies, and (**E**) diagnosed internal variability SAT anomalies, each averaged over 1993–2022. All SAT anomalies are defined relative to the 1961–1990 climatology.

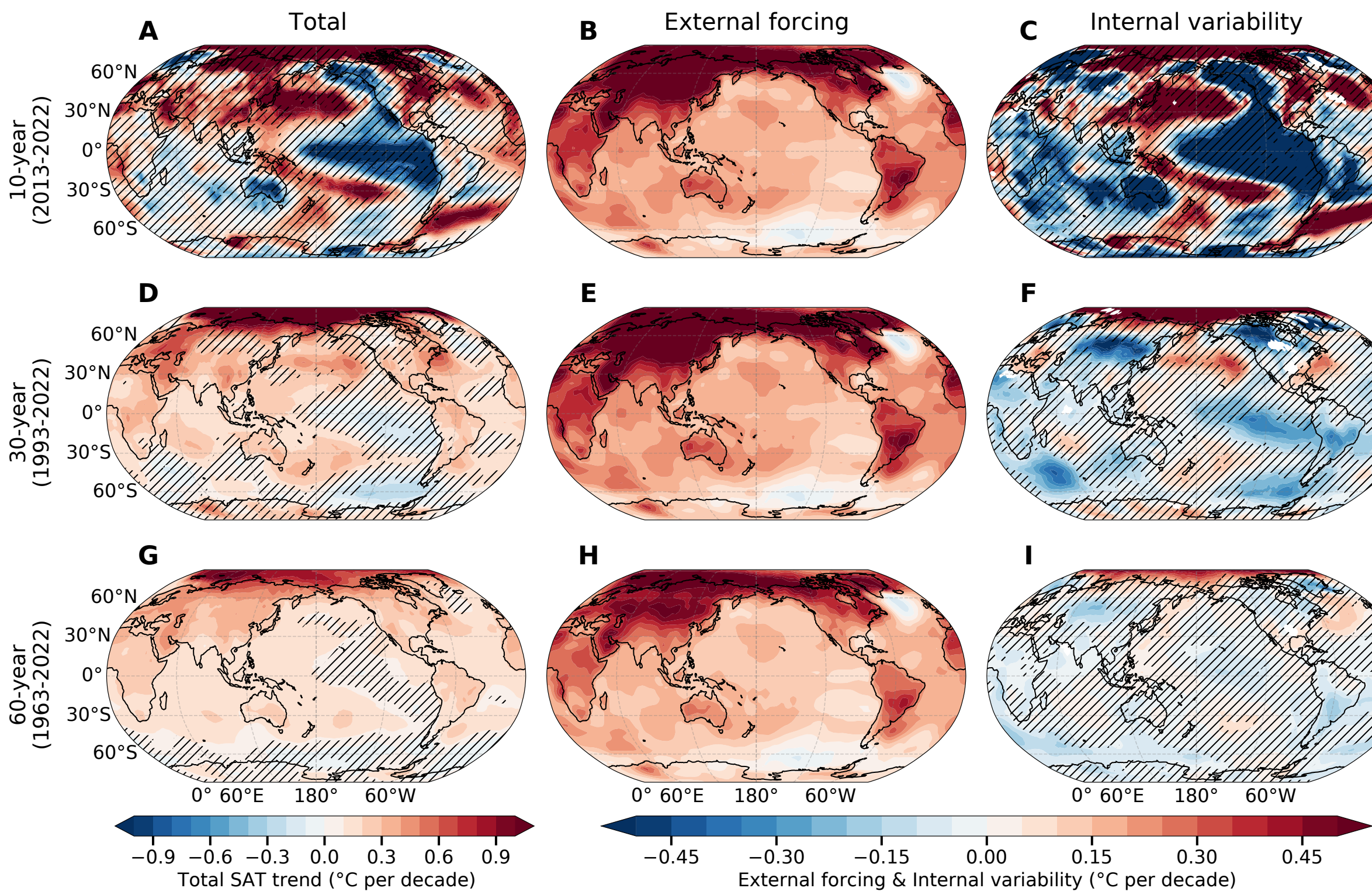

Fig. S3. Partitioning of observed SAT trend patterns into external forcing and internal variability. This figure follows the same methodology as Fig. 2, but is based on the NOAAGlobalTemp data.

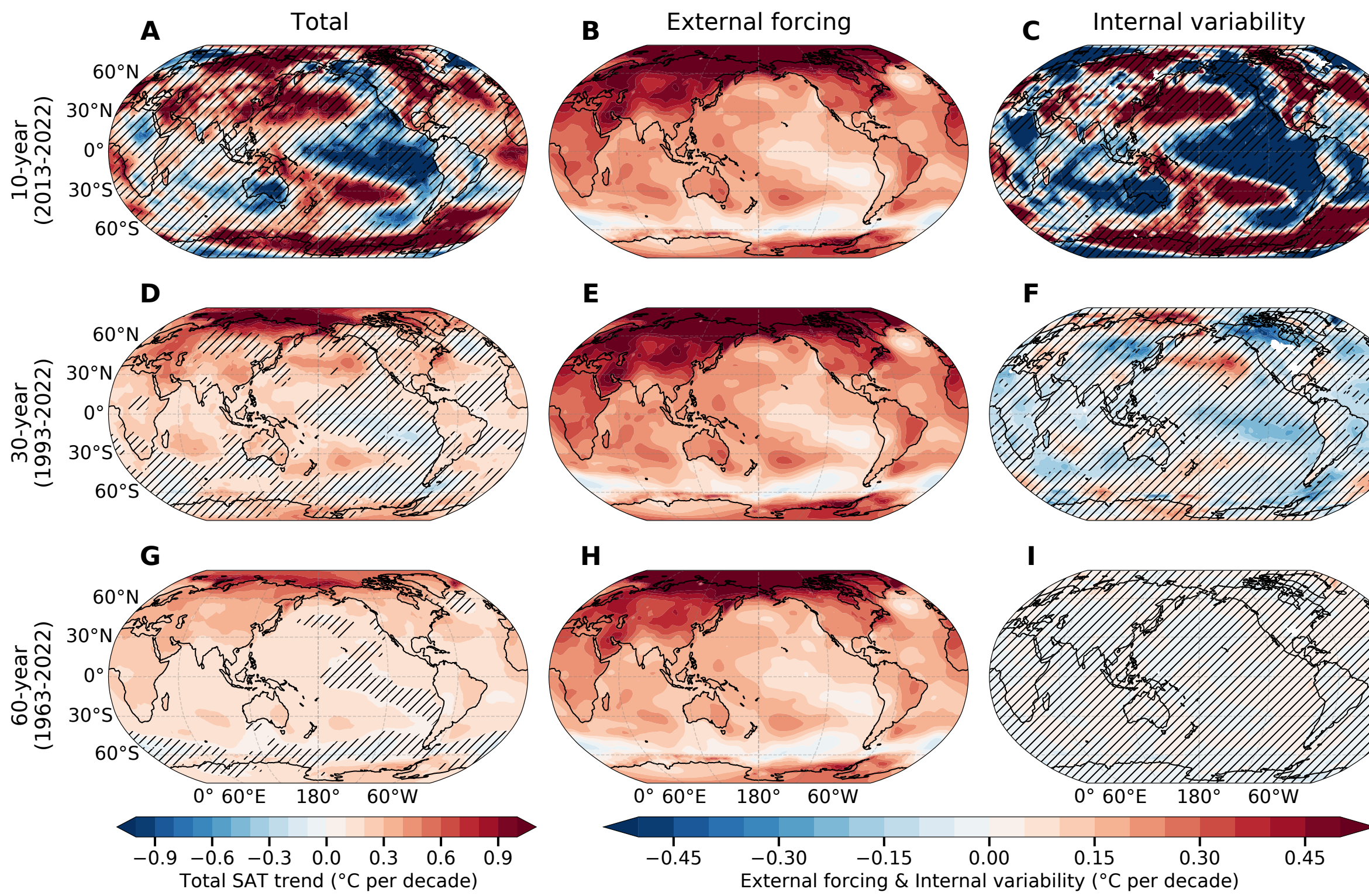

Fig. S4. Partitioning of observed SAT trend patterns into external forcing and internal variability. This figure follows the same methodology as Fig. 2, but is based on the Berkeley Earth Surface Temperature data.

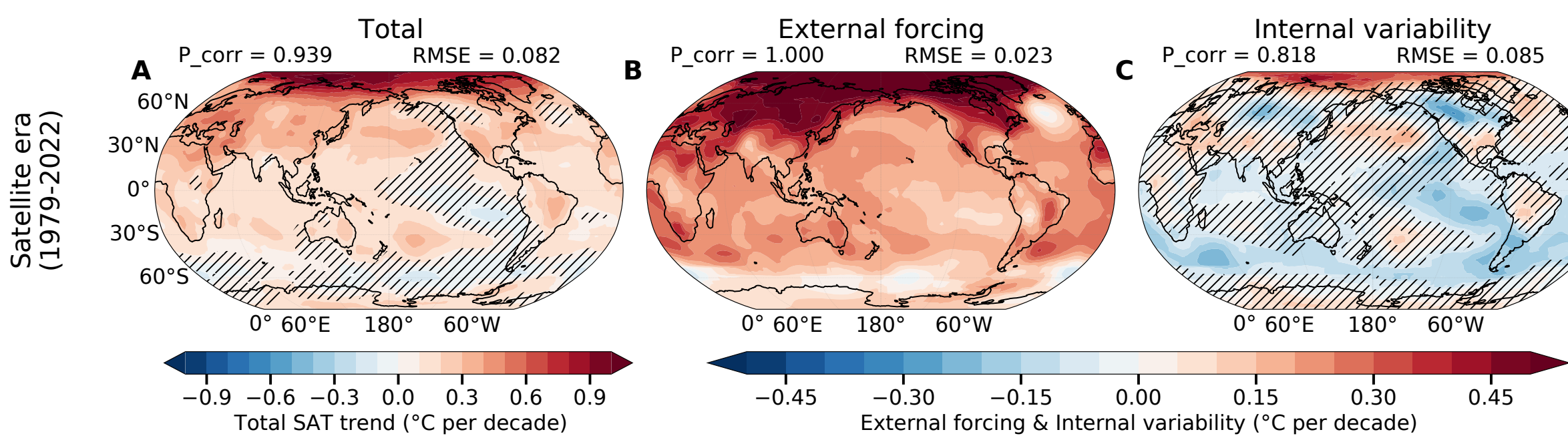

Fig. S5. Partitioning of observed SAT trend patterns into external forcing and internal variability for the Satellite era (1979–2022). (**A**) The total observed SAT trend, (**B**) the OBS–LPS diagnosed externally forced trend component, and (**C**) the residual (internal variability) trend component for the 44-year satellite-era period 1979–2022. Hatching denotes trends that are not significant at 95% confidence, as determined by the non-parametric Mann-Kendall trend test. The pattern correlation (P_{corr}) and root mean square error (RMSE) between the satellite-era trends (1979–2022) and the corresponding 30-year trends (1993–2022) are shown in each panel.

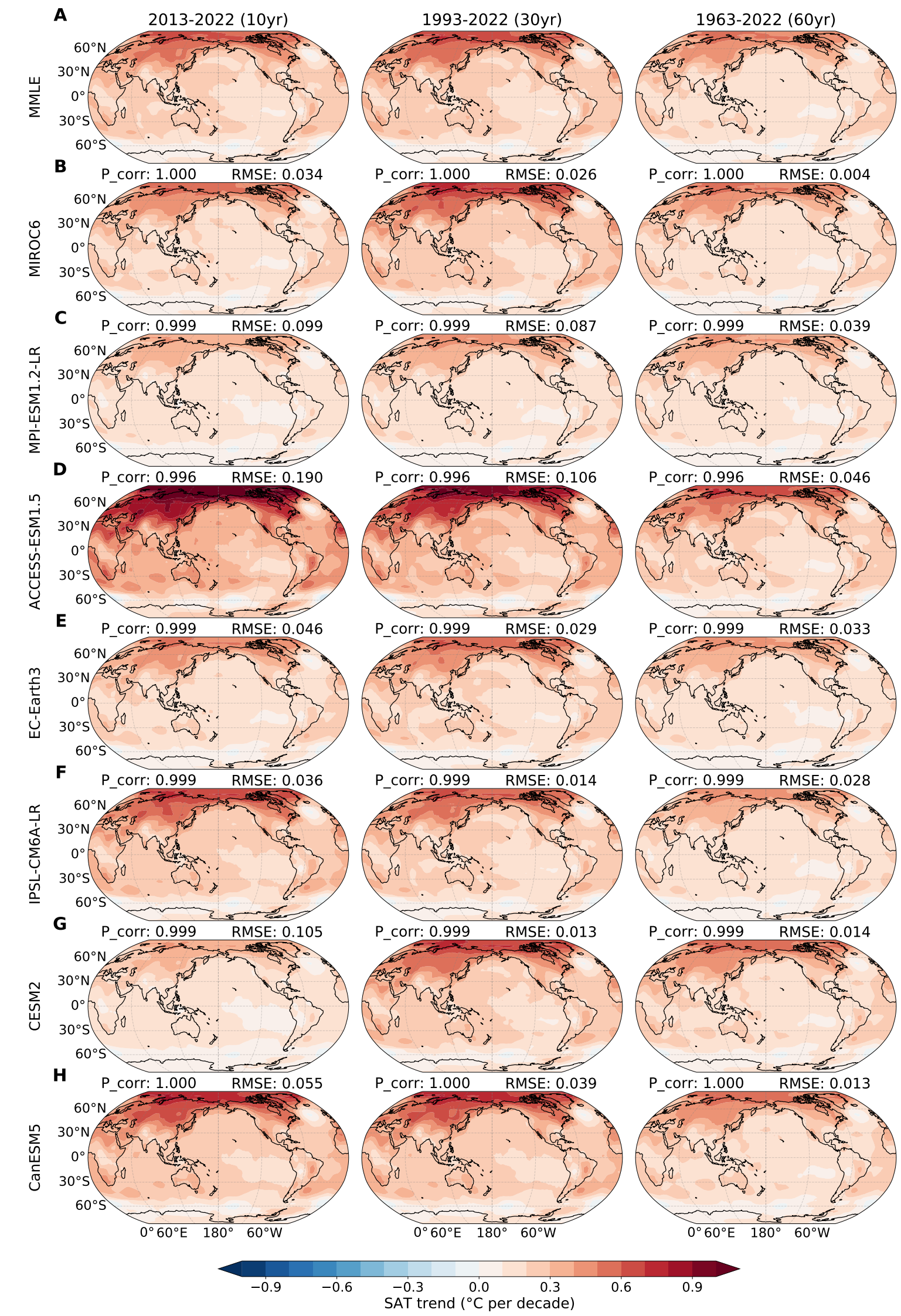

Fig. S6. Observed externally forced SAT trend patterns diagnosed using different GSAT predictors. Observed externally forced SAT trend patterns diagnosed with the OBS–LPS using alternative ensemble mean GSAT predictors: (**A**) MMLE, (**B–H**) individual LEs (MIROC6, MPI-ESM1.2-LR, ACCESS-ESM1.5, EC-Earth3, IPSL-CM6A-LR, CESM2, CanESM5; see labels). Columns (left to right) show trend periods of 10-year (2013–2022), 30-year (1993–2022), and 60-year (1963–2022). Values above the panels **B–H** give the pattern correlation and RMSE relative to the MMLE-GSAT-based result (panel **A**).

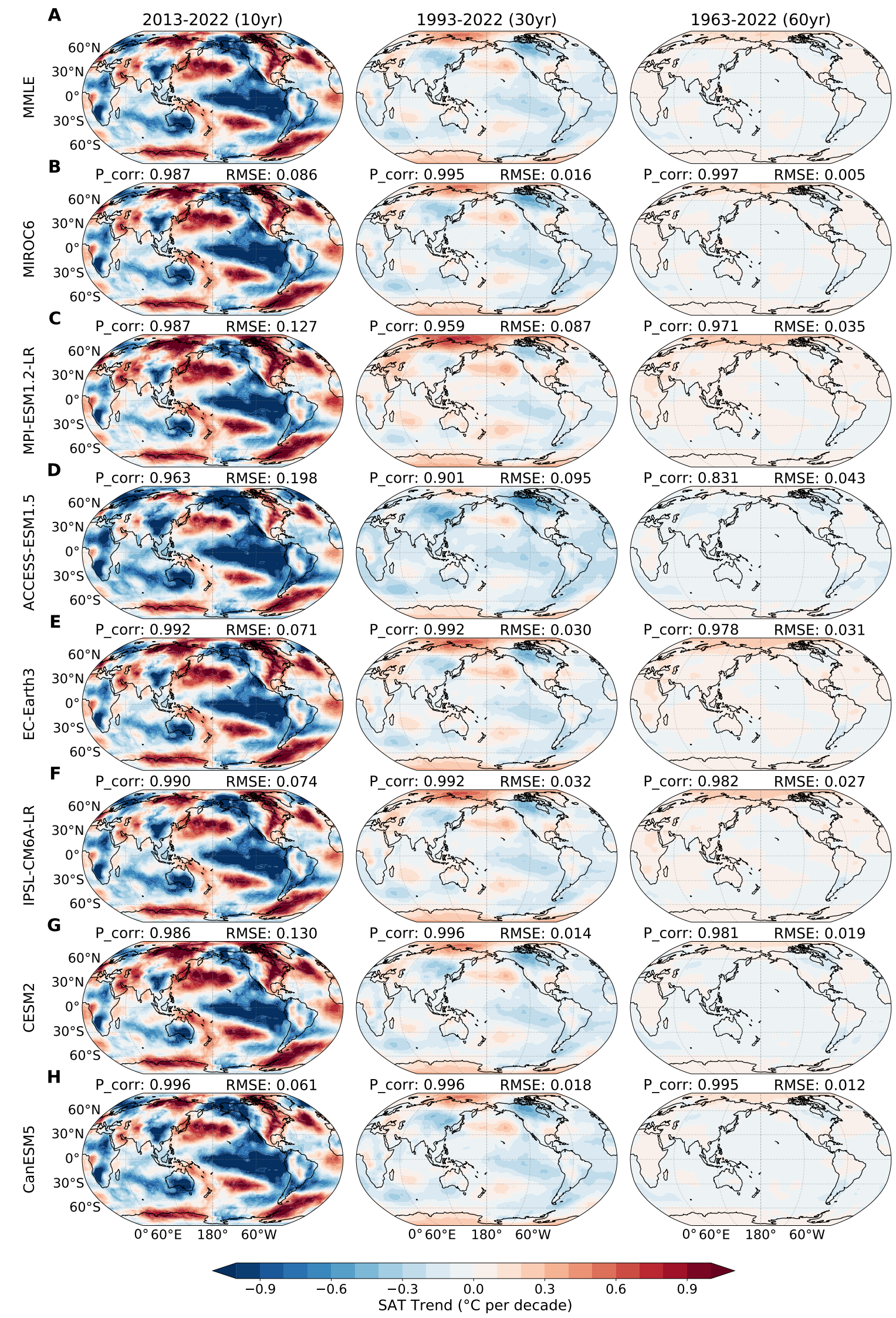

Fig. S7. Observed internal variability SAT trend patterns diagnosed using different GSAT predictors. Observed internal variability SAT trend patterns are diagnosed as OBS–LPS residuals using alternative ensemble mean GSAT predictors: (**A**) MMLE, (**B–H**) individual LEs (MIROC6, MPI-ESM1.2-LR, ACCESS-ESM1.5, EC-Earth3, IPSL-CM6A-LR, CESM2, CanESM5; see labels). Columns (left to right) show trend periods of 10-year (2013–2022), 30-year (1993–2022) and 60-year (1963–2022). Values above the panels **B–H** give the pattern correlation and RMSE relative to the MMLE-GSAT-based result (panel **A**).

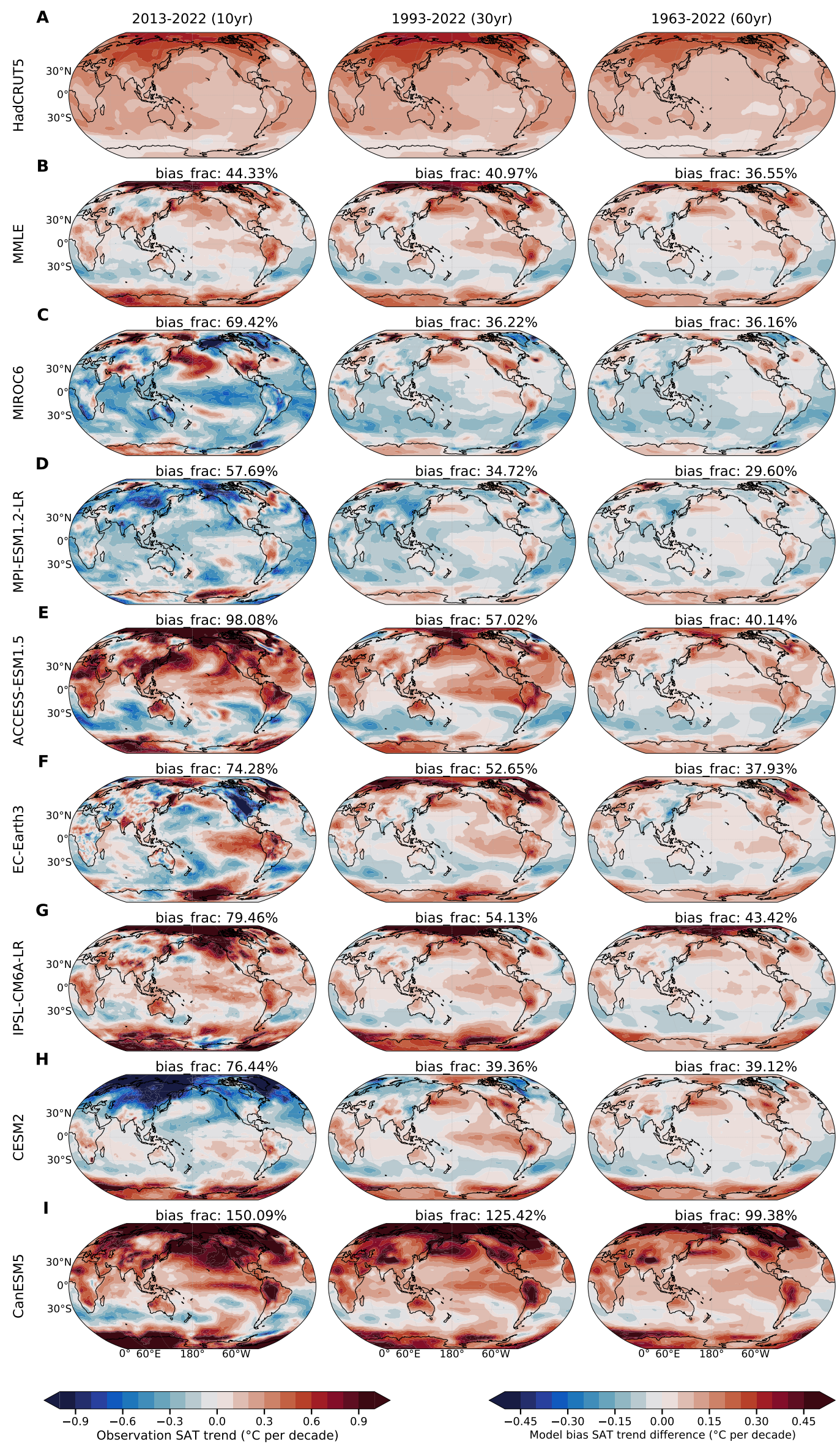

Fig. S8. Comparison of observed and simulated geographic patterns of externally forced annual mean SAT trends. (**A**) observed externally forced SAT trend patterns diagnosed with OBS–LPS. (**B-I**) Model–observation differences for MMLE, MIROC6, MPI-ESM1.2-LR, ACCESS-ESM1.5, EC-Earth3, IPSL-CM6A-LR, CESM2 and CanESM5 (see panel labels). Columns (left to right) show trend periods of the 10-year (2013–2022), 30-year (1993–2022) and 60-year (1963–2022). Percentages in **B-I** report the global bias-amplitude fraction, defined as the area-weighted RMS of the (LE–OBS) normalized by the area-weighted RMS of the observed pattern (×100%).

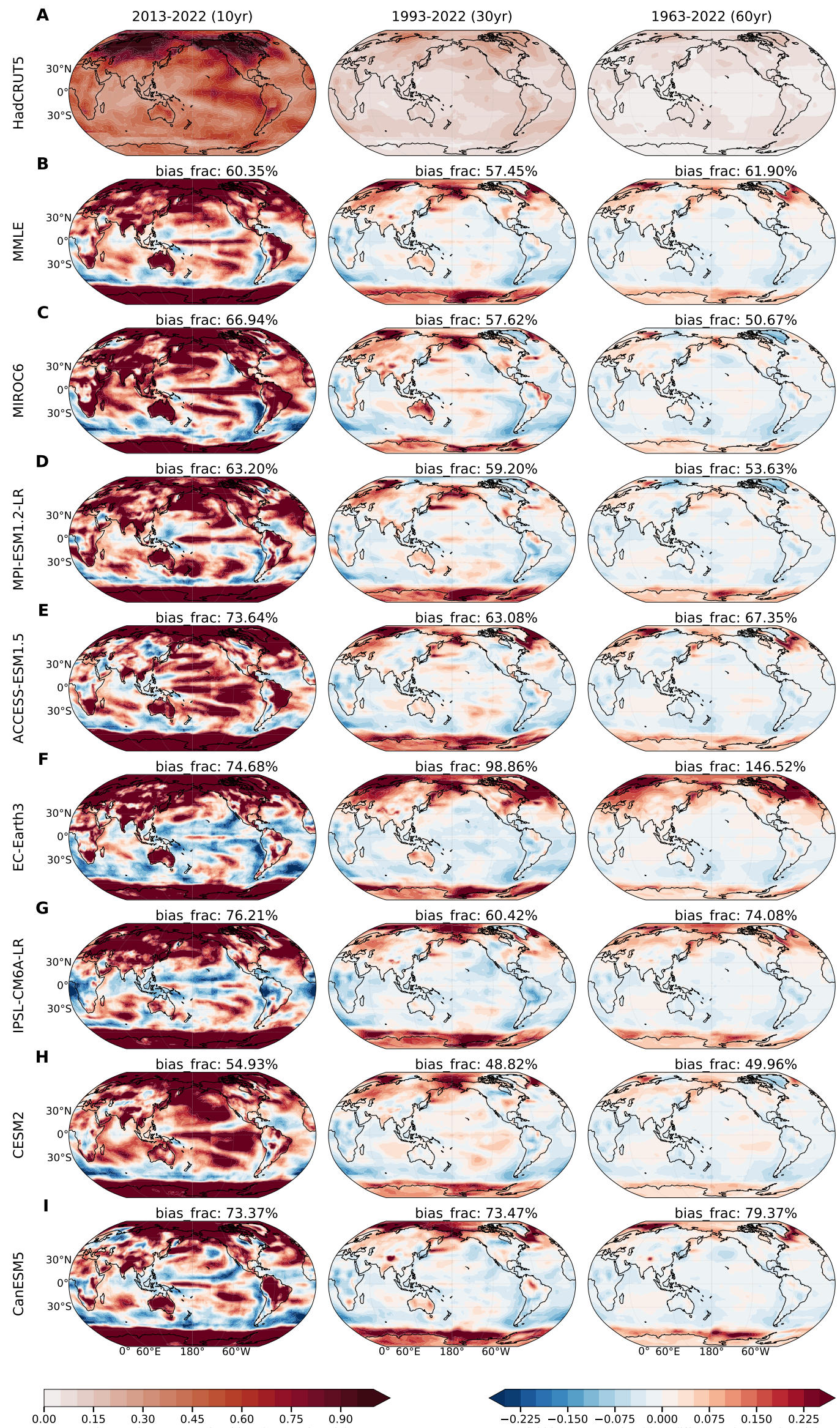

Fig. S9. Comparison of observed and simulated geographic patterns of internal variability in annual mean SAT trends. (**A**) Observed internal variability amplitude, defined as the standard deviation of OBS–LPS diagnosed internal variability SAT trends. (**B–I**) Model–observation differences in internal variability amplitude, computed as the ensemble-spread amplitude in each model or the MMLE minus the observed internal variability amplitude, for MMLE, MIROC6, MPI-ESM1.2-LR, ACCESS-ESM1.5, EC-Earth3, IPSL-CM6A-LR, CESM2, and CanESM5 (see panel labels). Columns (left to right) show trend periods of 10-year (2013–2022), 30-year (1993–2022) and 60-year (1963–2022). Percentages in **B–I** report the global bias-amplitude fraction, defined as the area-weighted RMS of the difference in internal variability amplitude (LE–OBS) normalized by the area-weighted RMS of the observed pattern (×100%).

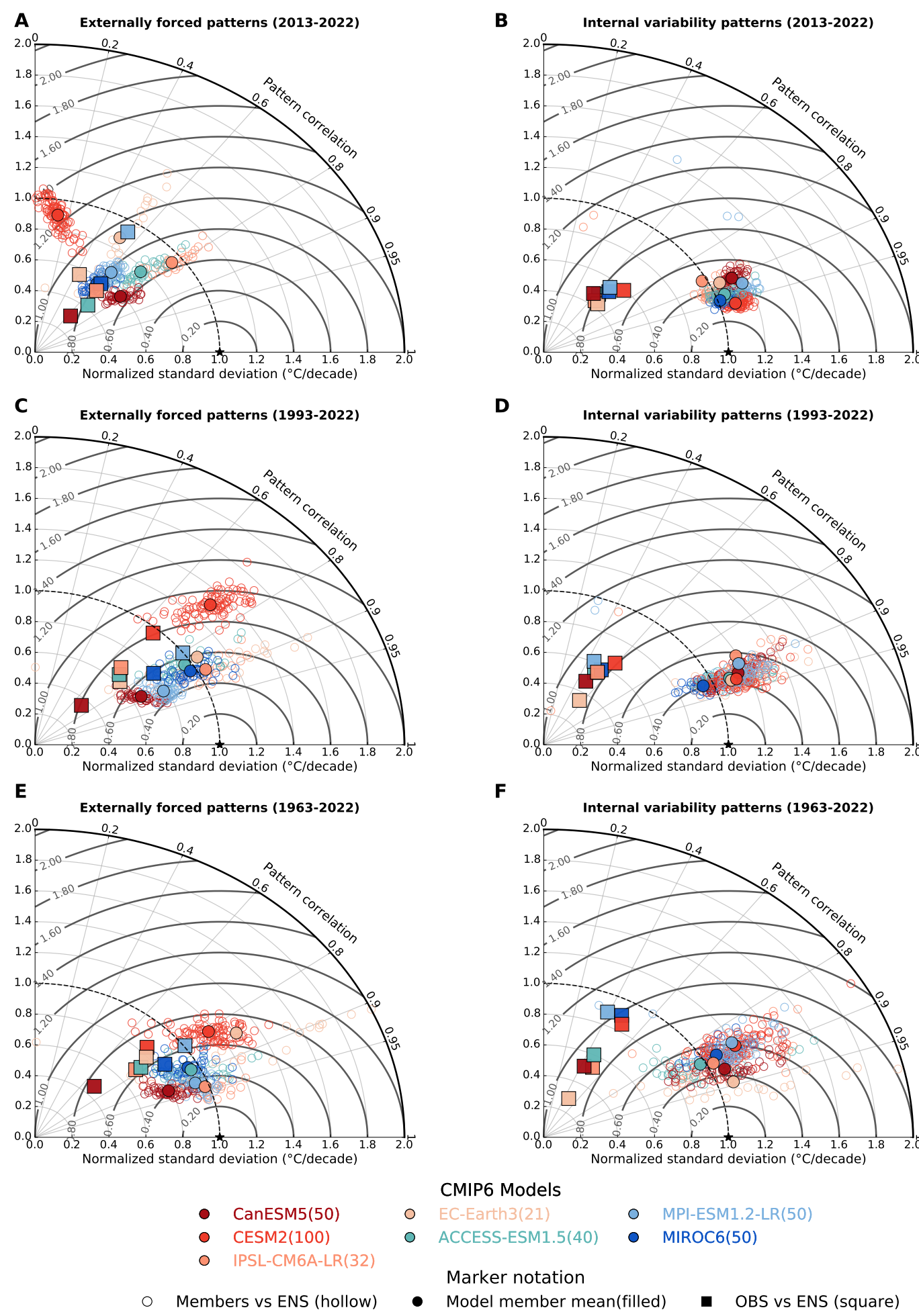

Fig. S10. LEs performance in characterizing observed SAT trend patterns for key periods. Normalized Taylor diagrams summarizing spatial pattern agreement using pattern correlation (azimuth), normalized standard deviation (radius; normalized by the reference), and normalized centered RMSE (nRMSE; black contours). (**A, C, E**) Externally forced SAT trend patterns for 10-year (2013–2022), 30-year (1993–2022), and 60-year (1963–2022), comparing member-wise OBS–LPS forced trends (hollow circles) with the corresponding LE ensemble mean forced trend pattern (reference). (**B, D, F**) As in (**A, C, E**), but for internal variability, comparing member-wise OBS–LPS residual trend patterns with the LE internal variability amplitude pattern defined as the spatial standard deviation of member trends (ensemble spread; reference). Filled circles denote the ensemble-member mean statistics for each LE. Squares denote the agreement between the observed forced/internal patterns and the corresponding LE/MMLE references (see Materials and Methods).

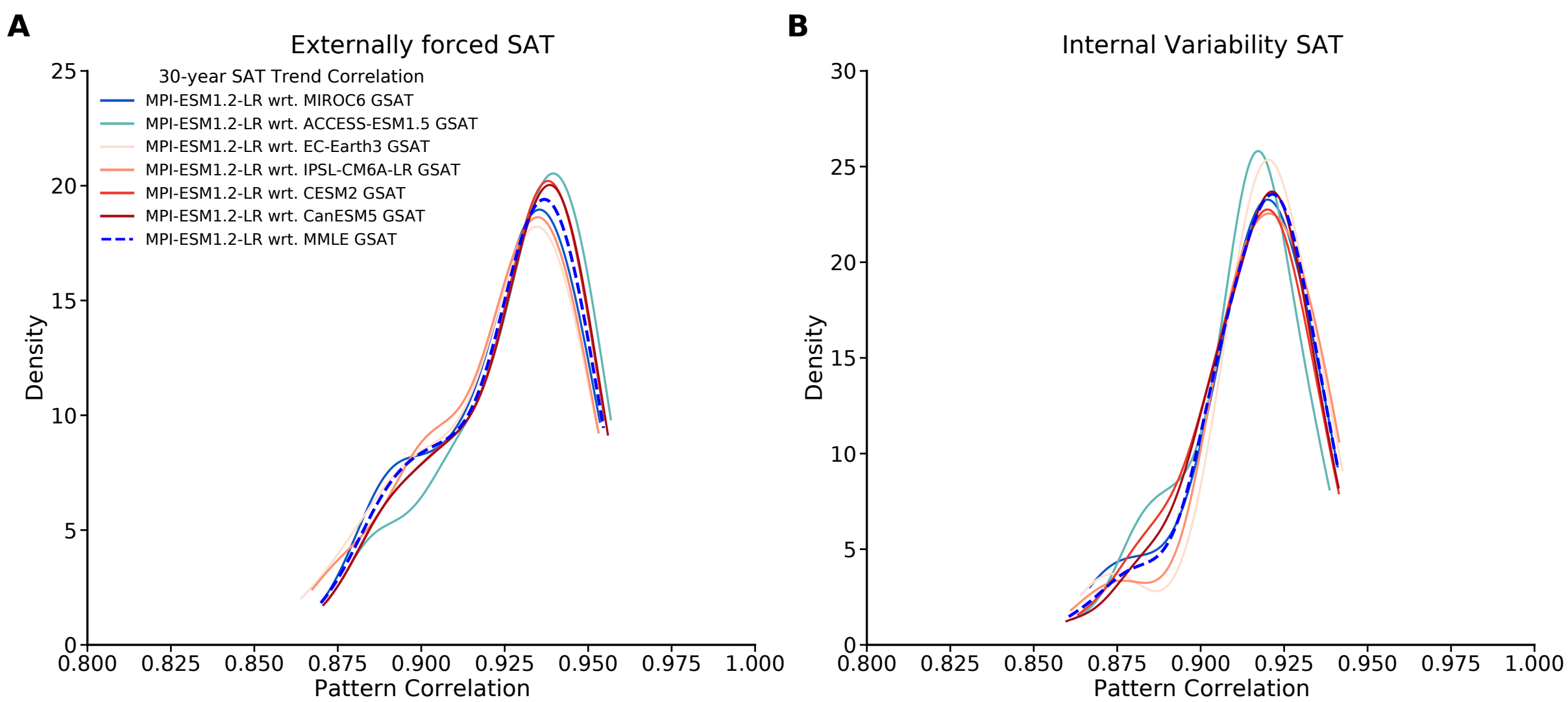

Fig. S11. Cross-model validation of OBS–LPS using MPI-ESM1.2-LR members as pseudo-observations (30-year trends, 1993–2022). (**A**) Pattern correlations between forced SAT trend patterns from individual MPI-ESM1.2-LR members and the MPI-ESM1.2-LR ensemble-mean forced pattern, using GSAT predictors from six other large ensembles (MIROC6, ACCESS-ESM1.5, EC-Earth3, IPSL-CM6A-LR, CESM2 and CanESM5) (solid colored lines) and from the MMLE (blue dashed line). (**B**) Corresponding pattern correlations for internal variability (standard deviation) SAT patterns versus the MPI-ESM1.2-LR ensemble spread pattern.

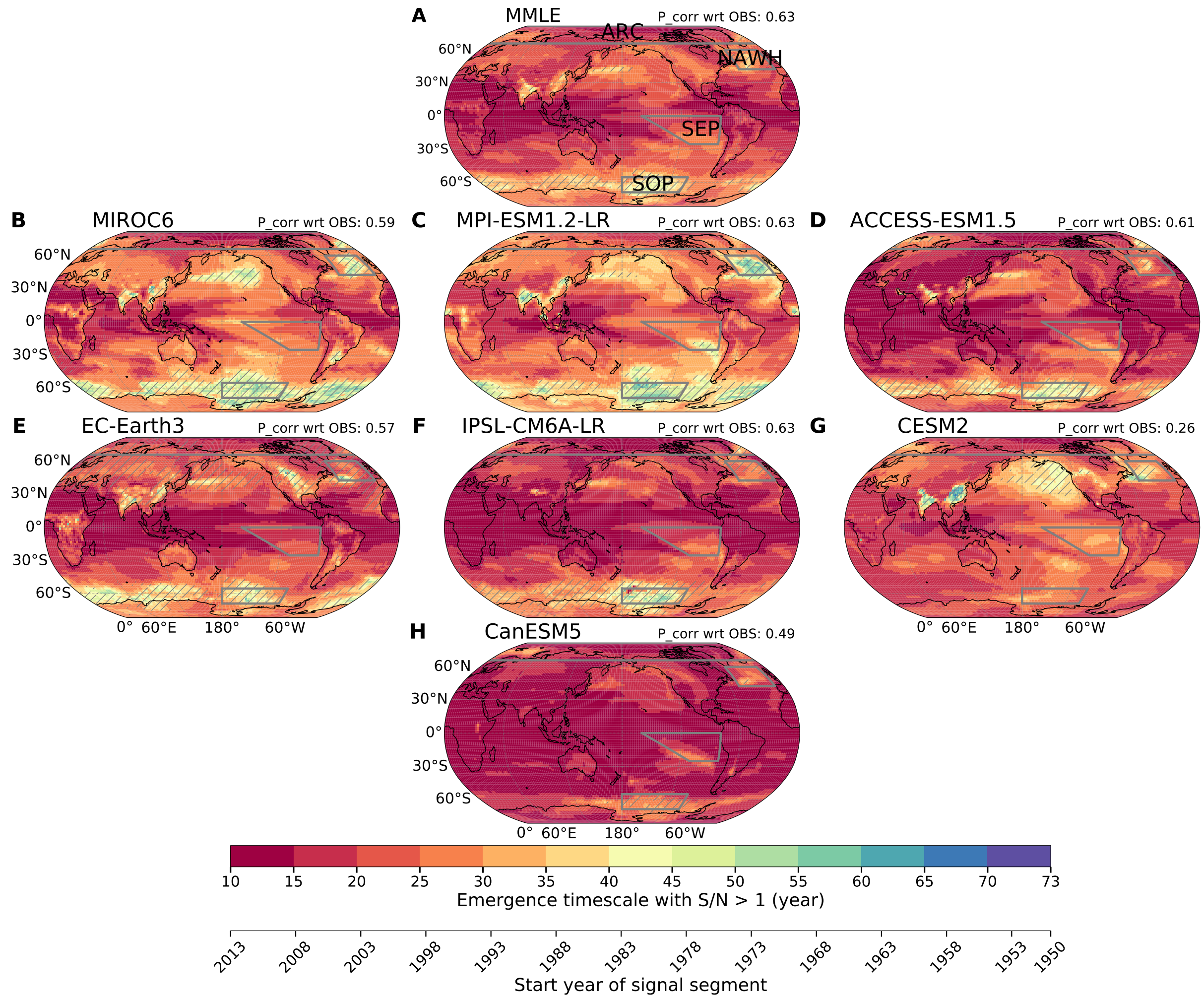

Fig. S12. Emergence timescale of simulated SAT trends during the historical period (1950–2022). Emergence timescale of simulated SAT trends from (**A**) MMLE and from individual LE: (**B**) MIROC6, (**C**) MPI-ESM1.2-LR, (**D**) ACCESS-ESM1.5, (**E**) EC-Earth3, (**F**) IPSL-CM6A-LR, (**G**) CESM2, and (**H**) CanESM5. Colored shading reflects the emergence timescale (years), while the overlaid secondary x-axis indicates the corresponding start year of each signal segment. Hatching indicates grid points where fewer than 85% of individual ensemble members satisfy the monotonic emergence criterion. For the MMLE, hatching marks grid points where fewer than four of the seven contributing models (i.e., <66%) meet the monotonic emergence test. Numbers in the upper-left corners give the spatial pattern correlation between each LE and the observed emergence timescale map. Grey-outlined boxes demarcate the four key regions referenced in the main text Fig. 5.

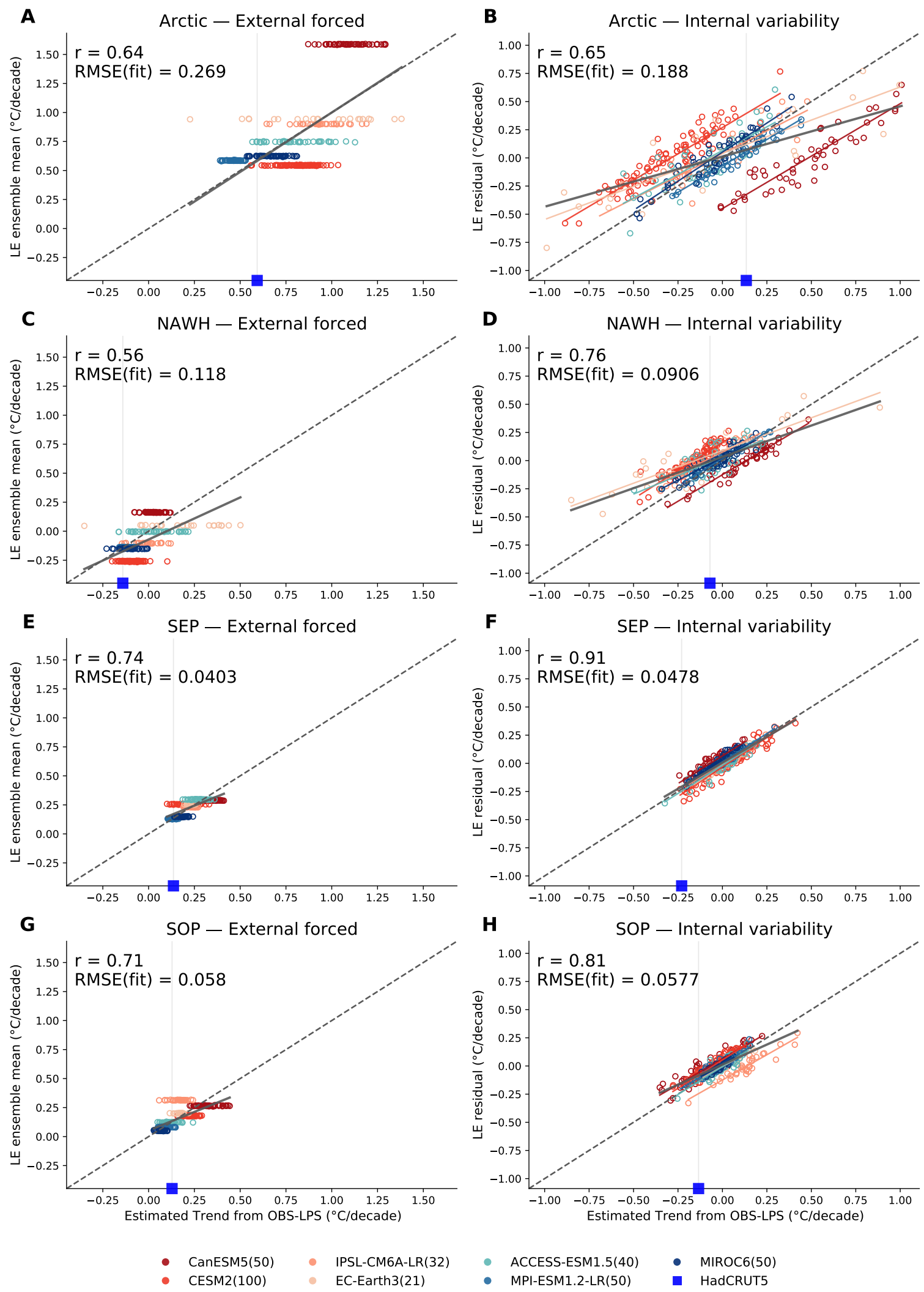

Fig. S13. Regional SAT trend estimates from the OBS–LPS framework versus large-ensemble benchmarks (1993–2022). (**A–B**) Arctic, (**C–D**) North Atlantic warming hole (NAWH), (**E–F**) Southeastern Pacific (SEP), and (**G–H**) Southern Ocean Pacific sector (SOP); regions are defined in the main text Fig. 4. For each region, the left panel compares the OBS–LPS diagnosed externally forced trend with the corresponding LE ensemble mean forced trend, and the right panel compares the OBS–LPS diagnosed internal variability component with the corresponding LE residual trend. Open circles denote individual realizations from the seven CMIP6 large ensembles (colors indicate models). Squares indicate the observational estimates (with the grey shading showing the spread across observational datasets, where applicable). The grey dashed diagonal is the 1:1 line in all panels.

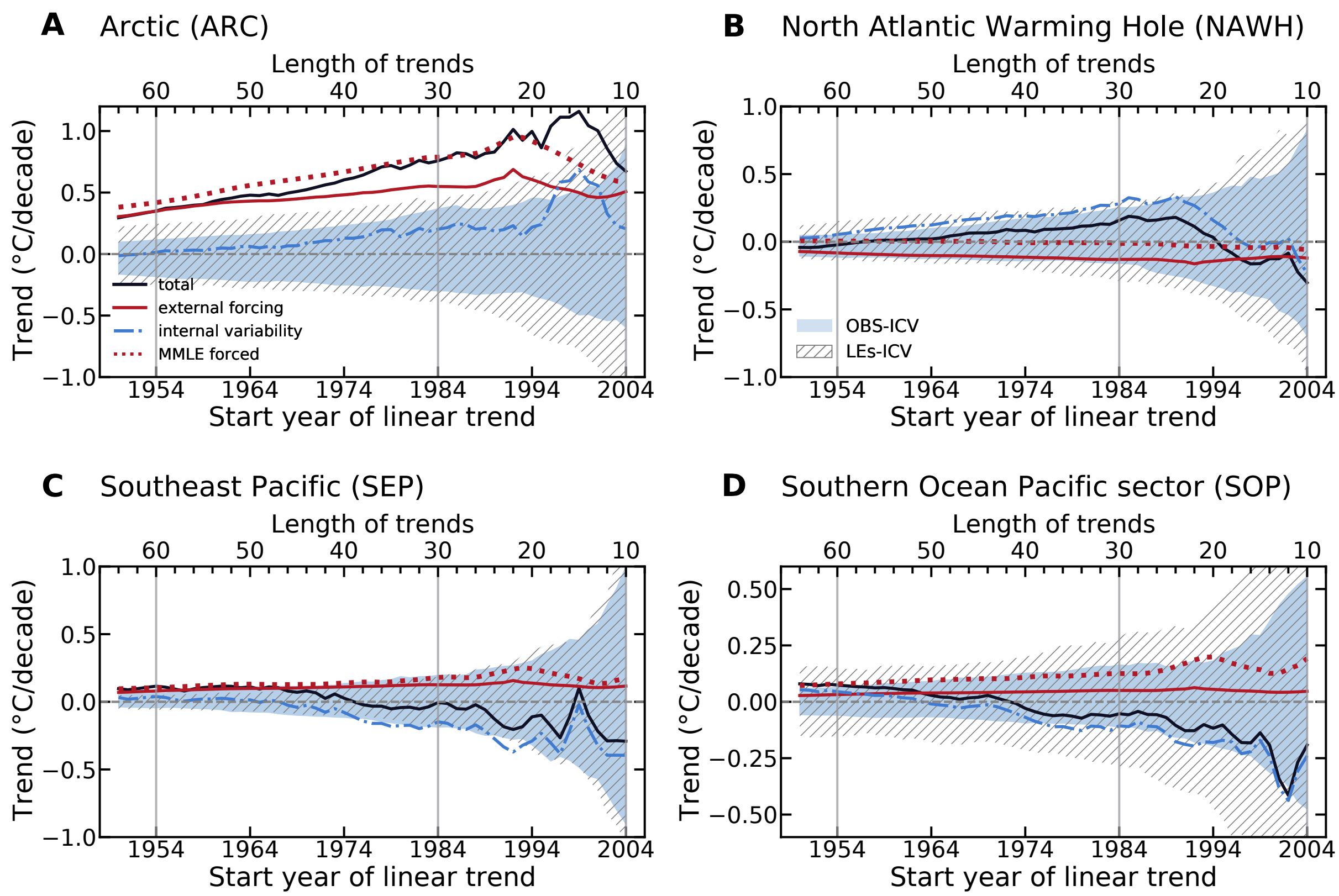

Fig. S14. Evolution of total, externally forced, and internal variability SAT trends in four key regions (1950–2013, anchored at 2013). (**A**) Arctic (ARC), (**B**) North Atlantic warming hole (NAWH), (**C**) Southeastern Pacific (SEP), and (**D**) Southern Ocean Pacific sector (SOP); regions are defined in the main text Fig. 4 (Materials and Methods). For each panel, regional-mean linear trends are computed over backwards-looking windows ending in 2013, with the start year advanced annually from 1950 to 2004 (top axis indicates the corresponding trend length). Black solid lines show total SAT trends; red solid lines show OBS–LPS diagnosed externally forced trends; and blue dash-dot lines show the diagnosed internal variability component. Light blue shading denotes the 5th–95th percentile range of observed internal variability estimates, derived from all overlapping trend samples within 1850–2022. The red dotted line shows the MMLE-simulated forced trend. Hatched shading shows the MMLE internal variability 5th–95th percentile range diagnosed from the seven CMIP6 large ensembles using an ensemble–residual approach (text S1). Grey vertical lines mark the specific trend intervals highlighted in main text Figs. 2–4.

Table S1: **CMIP6 single model initial-condition large ensembles used in this study.** Scenarios are listed up to 2022; ECS is effective climate sensitivity (°C); *N* is the number of ensemble members.

**Model**	**Variable / Scenarios**	**ECS**	**N**	**Institution**	**Source**
ACCESS-ESM1.5	tas; Historical; SSP2-4.5	3.9	40 (r1i1p1f1)	Commonwealth Scientific and Industrial Research Organization; Bureau of Meteorology (Australia)	Ziehn et al., 2020 (*67*)
CanESM5	tas; Historical; SSP2-4.5	5.6	50 (r1i1p1f1, r1i1p2f1)	Canadian Centre for Climate Modelling and Analysis (CCCma), Canada	Swart et al., 2019 (*68*)
CESM2	TREFHT; Historical; SSP3-7.0	5.3	100 (CMIP6 protocol and CMIP6 SMBB protocol)	National Center for Atmospheric Research (NCAR), Climate and Global Dynamics Laboratory, Boulder, USA	Rodgers et al., 2021 (*73*)
EC-Earth3	tas; Historical; SSP2-4.5	4.3	21 (r1i1p1f1)	EC–Earth Consortium (27 European institutes)	Döscher et al., 2022 (*69*)
IPSL-CM6A-LR	tas; Historical; SSP2-4.5	4.7	32 (r1i1p1f1)	Institut Pierre–Simon Laplace Climate Modelling Centre, France	Boucher et al., 2020 (*70*)
MIROC6	tas; Historical; SSP2-4.5	2.6	50 (r1i1p1f1)	JAMSTEC; AORI (The University of Tokyo); NIES; RIKEN CCS, Japan	Tatebe et al., 2019 (*71*)
MPI-ESM1.2-LR	tas; Historical; SSP2-4.5	2.8	50 (r1i1p1f1)	Max Planck Institute for Meteorology, Germany	Olonscheck et al., 2023 (*72*)
